# Supplementary material for: A bacterial effector counteracts host autophagy by promoting degradation of an autophagy component
Source: EMBO J. 2022 May 27;41(13):e110352. doi: 10.15252/embj.2021110352 (PMC9251887; doi:10.15252/embj.2021110352)
Supplement: Supplementary file 1 — Appendix [file EMBJ-41-e110352-s006.pdf]

Appendix

Appendix Figure S1: Silencing of *ATG7* in *N. benthamiana* plants abolishes autophagosome formation and Xcv blocks autophagy at 6hpi.
Appendix Figure S2: Virus induced gene silencing of *ATG7* in *N. benthamiana* is beneficial for Xcv.
Appendix Figure S3: Suppression of autophagy is enhanced by T3Es**.**
Appendix Figure S4: Screening for Xanthomonas T3Es with altered autophagic flux.
Appendix Figure S5: XopL contributes to Xcv virulence.
Appendix Figure S6: Joka2 bodies are induced during Xcv infection in a XopL-dependent manner.
Appendix Figure S7: Transgenic *A. thaliana* GFP-XopL plants display defects in autophagic degradation.
Appendix Figure S8: SH3P2 is conserved in different plant species.
Movie EV1: XopL/SH3P2 puncta are mobile.
Appendix Figure S9: Silencing of SH3P2 in *N. benthamiana* perturbs autophagy.
Appendix Figure S10: Gene expression of SH3P2 is induced by XopL and XopL-mediated degradation is due to post-transcriptional degradation events.
Appendix Figure S11: RFP-XopL ΔE3 co-localizes with and is unable to ubiquitinate SH3P2-GFP.
Appendix Figure S12: XopL is degraded in the vacuole.
Appendix Figure S13: Virus-induced gene silencing of *Joka2* in *N. benthamiana* plants.
Appendix Figure S14: XopL in planta ubiquitination is enhanced by the presence of AIMp.
Appendix Figure S15: XopL is ubiquitinated *in planta* and undergoes self-ubiquitination.
Appendix Figure S16: Characterization of $XopL_{K191A}$ variant i*n vitro* and *in planta*.
Appendix Figure S17: XopL ΔE3 is degraded by autophagy.
Appendix Figure S18: Model illustrating the function of XopL
Appendix Figure S19: XopL K191 residue is highly conserved through the Xanthomonas genus
Appendix Table S1: Primers used in manuscript.

Appendix Figure S1

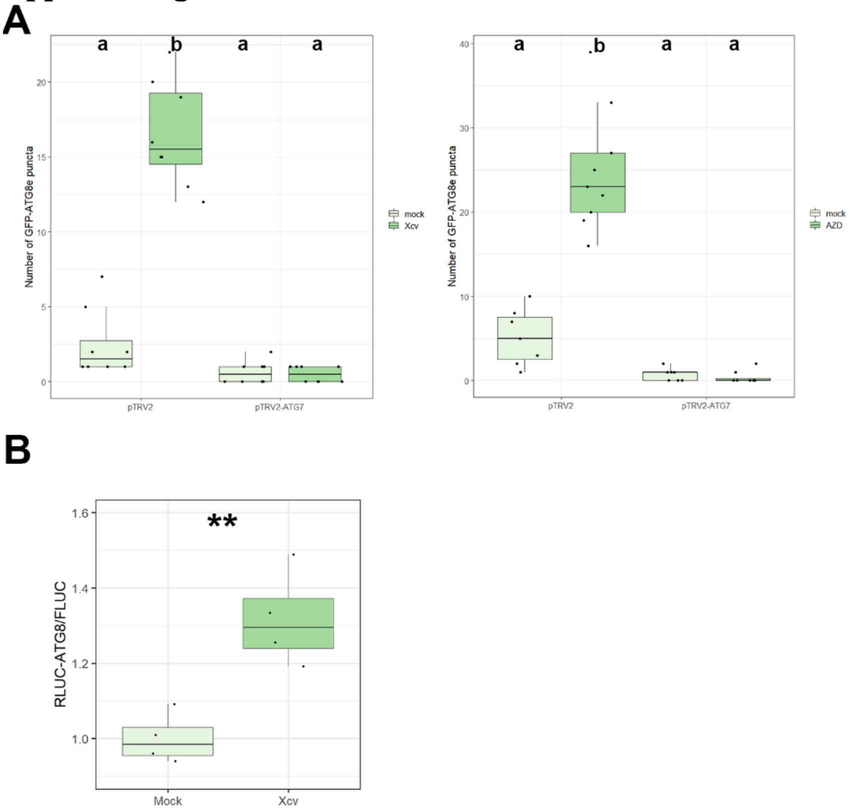

Fig. S1: Silencing of *ATG7* in *N. benthamiana* plants abolishes autophagosome formation and Xcv blocks autophagy at 6hpi. GFP-ATG8e-labeled puncta were quantified from plants silenced for *ATG7* (pTRV2-ATG7) infected with mock or Xcv *ΔxopQ* at 6hpi in the presence or absence of ConA, and of AZD. Puncta were calculated from z-stacks (X) of *n*=12 individuals using ImageJ. Different letters indicate statistically significant different groups ($P < 0.05$) as determined by one way ANOVA.

(B) RLUC-ATG8a or RLUC-NBR1 constructs were coexpressed with internal control FLUC in *N. benthamiana*. *Xcv ΔxopQ* or infiltration buffer (mock) was co-infiltrated with Agrobacteria containing the respective constructs. RLUC and FLUC activities were simultaneously measured in leaf extracts at 8 h post- infiltration using the dual-luciferase system. Values represent the ratio of RLUC-ATG8a and FLUC activities (n=4). Statistical significance (***$P < 0.001$) was revealed by Student's *t*-test. The experiment was repeated 3 times with similar results.

Appendix Figure S2

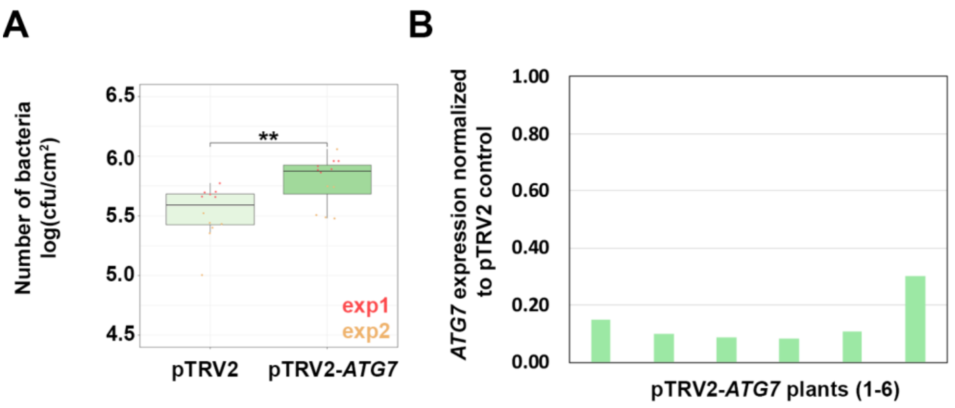

Fig. S2: Virus induced gene silencing of *ATG7* in *N. benthamiana* is beneficial for Xcv.

(A) Growth of Xcv *ΔxopQ* in *N. benthamiana* plants silenced for *ATG7* (pTRV2-*ATG7*) compared to control plants (pTRV2). Leaves were dip-inoculated with a bacteria suspension at OD_{600} = 0.2 and bacteria were quantified at 6 dpi. Data represent the mean SD (n = 6). Significant differences were calculated using Student's *t-test* and are indicated by **, $P < 0.01$. The experiment was repeated twice with similar trends. Red and yellow data points represent repeats of the experiment.

(B) qRT-PCR analysis of *ATG7* mRNA levels in silenced *N. benthamiana* plants. *Actin* expression was used to normalize the expression value in each sample, and relative expression values were determined against the mean expression in pTRV2 (control) plants**.**

Appendix Figure S3

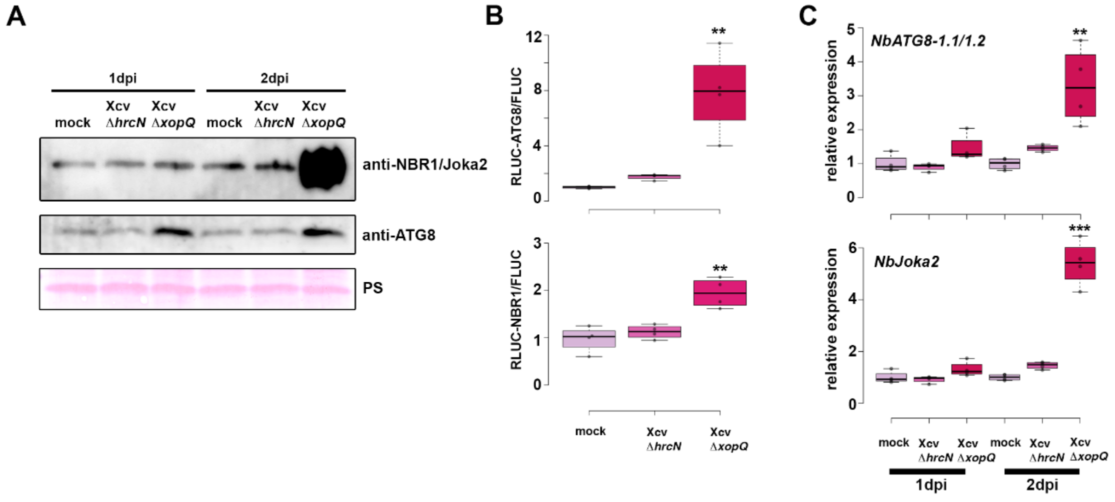

Fig. S3: Suppression of autophagy is enhanced by T3Es.

(A) Immunoblot analysis of NBR1 and ATG8 protein levels in Xcv *ΔxopQ, ΔhrcN* or mock infected *N. benthamiana* plants at 1 and 2dpi. Ponceau Staining (PS) served as a loading control. The experiment was repeated twice with similar results.

(B) Autophagic flux determined by quantitative dual-luciferase assay. RLUC-ATG8a or RLUC-NBR1 constructs were coexpressed with internal control FLUC in *N. benthamiana*. Xcv *ΔxopQ* and *ΔhrcN* were respectively co-infiltrated with Agrobacteria containing the luciferase constructs. *Renilla* and *Firefly* luciferase activities were simultaneously measured in leaf extracts at 48 h post- infiltration using the dual-luciferase system. Values represent the ratio of RLUC-ATG8a and FLUC activities normalized to mock (n=4). Statistical significance

(**P<0.01) was revealed by Student's *t*-test. The experiment was repeated 2 times with similar results.

(C) RT-qPCR analysis of *NbATG8-1.1/2* and *NbJoka2* transcript levels upon challenge of *N. benthamiana* plants with Xcv *ΔxopQ* and *ΔhrcN* for 1 and 2 dpi compared to mock infected plants. Values represent expression relative to mock control of respective time point and were normalized to *actin*. Statistical significance (*** P < 0.001) was revealed by Student's *t*-test.

Appendix Figure S4

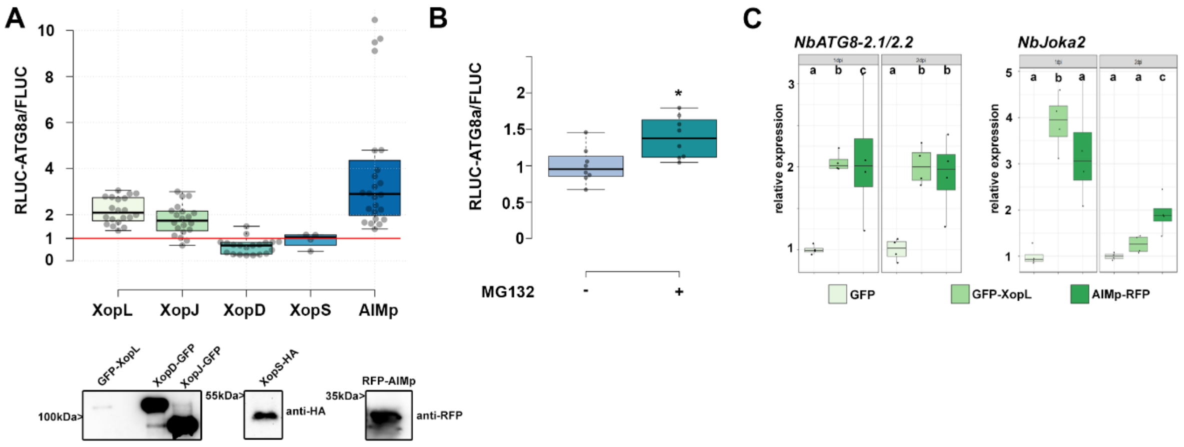

Fig. S4: Screening for Xanthomonas T3Es with altered autophagic flux.

(A) RLUC-ATG8a constructs were coexpressed with internal control FLUC in *N. benthamiana*. GFP-XopL, XopJ-GFP, XopD-GFP and XopS-HA were co-infiltrated with Agrobacteria carrying the RLUC-ATG8a and FLUC constructs. Renilla and Firefly luciferase activities were simultaneously measured in leaf extracts at 48 h post- infiltration using the dual-luciferase system. Values represent the ratio of RLUC-ATG8a to FLUC activity normalized to GFP control (XopL, XopJ, XopD, AIMp; n=20; XopS n=4). Expression of T3Es and RFP-AIMp were verified with the indicated antibodies.

(B) RLUC-ATG8a constructs were coexpressed with internal control FLUC in *N. benthamiana*. Plants were treated with MG132 for 6 hours prior measurement. Values represent the ratio of RLUC-ATG8a to FLUC activity normalized to vector control (n=8). Statistical significance (* P < 0.5) was revealed by Student's *t*-test.

(C) RT-qPCR analysis of *NbATG8-2.1/2* and *NbJoka2* transcript levels upon Agrobacteria-mediated transient expression of GFP, GFP-XopL or AIMp for 1 and 2 dpi. Values represent expression relative to GFP control of respective time point and were normalized to *actin*. Statistical significantly different groups are denoted by different letters, as calculated using Kruskal-Wallis rank sum test (P<0.05).

Appendix Figure S5

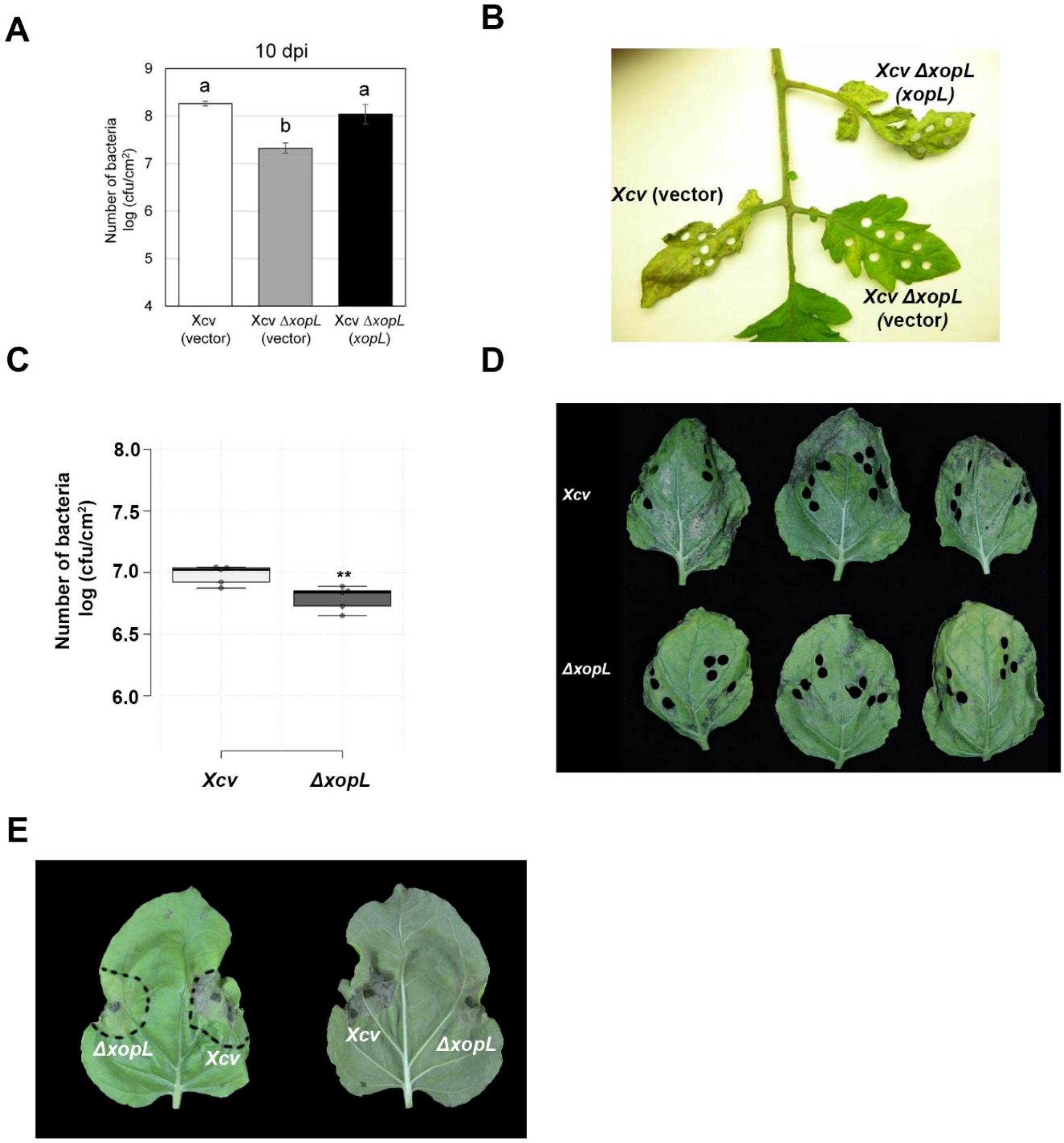

Fig. S5: XopL contributes to Xcv virulence.

(A) Growth of Xcv 85-10 (vector) (white bar), Xcv 85-10 Δ*xopL* (vector) (grey bar), and Xcv 85-10 Δ*xopL* (*xopL*) (black bar) strains in tomato VF36 leaves. Leaves were dipped in a 2 x 10^8 CFU/mL suspension of bacteria. The number of bacteria in each leaf was quantified at 10 dpi. Data points represent mean $\log_{10}$ colony-forming units per cm^2 ± SD of three plants. Different letters above bars indicate statistically significant (Tukey's honestly significant difference (HSD) test, $P < 0.05$) differences between samples. Vector = pBBR1MCS-2.

(B) Delayed disease symptom development in tomato leaves inoculated with *Xcv* or *Xcv* Δ*xopL*. Tomato leaves inoculated with strains described in (A) were photographed at 14 dpi.

(C) Growth of Xcv 85-10, Xcv 85-10 Δ*xopL* strains in *roq1 N. benthamiana* leaves. Leaves were dipped in a 2 x 10^8 CFU/mL suspension of bacteria. The number of bacteria in each leaf was quantified at 10 dpi (n = 5). Significant differences were calculated using Student's *t-test* (**, $P < 0.01$). The experiment was repeated twice with similar trends.

(D) Delayed disease symptom development in *roq1 N. benthamiana* leaves dip-inoculated with *Xcv* or *Xcv* Δ*xopL*. *N. benthamiana roq1* leaves inoculated with strains described in (C) were photographed at 10 dpi.
(E) Delayed symptom development in *roq1 N. benthamiana* leaves inoculated with *Xcv* or *Xcv* Δ*xopL*. Leaves were syringe-inoculated with OD_{600}=0.2 and photographed at 3 dpi.

Appendix Figure S6

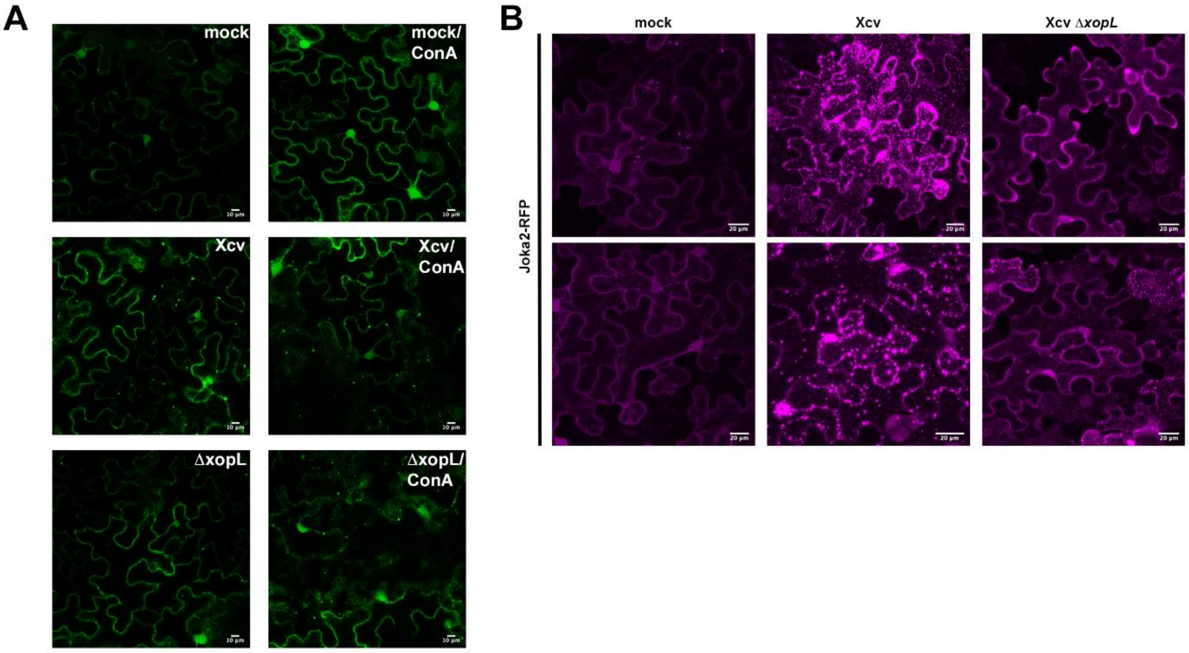

Fig. S6: Joka2 bodies are induced during Xcv infection in a XopL-dependent manner.
(A) GFP-ATG8e-labeled autophagosomes imaged from *N. benthamiana* plants infected with mock, Xcv or *Xcv* Δ*xopL* at 2 dpi in the presence or absence of ConA (bars = 10 μm).
(B) RFP-Joka2 labelled puncta or aggregates upon challenge of *N. benthamiana* leaves with mock, *Xcv* or *Xcv* Δ*xopL* infection at 1dpi.

Appendix Figure S7

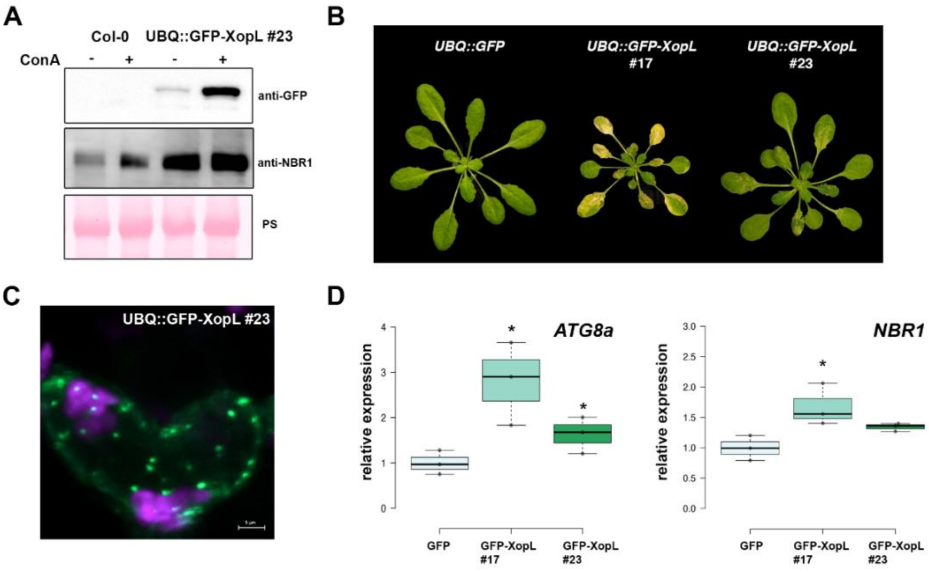

Fig. S7: Transgenic *A. thaliana* GFP-XopL plants display defects in autophagic degradation

(A) Immunoblot analysis of NBR1 protein levels in transgenic UBQ::GFP-XopL plants or Col-0. Plants were treated with concanamycin A (ConA) for 6 hours. Expression of GFP-XopL was verified with an anti-GFP antibody. Ponceau S staining serves as a loading control.

(B) 5 weeks old *A. thaliana* plants expressing UBQ::GFP-XopL develop an early senescence phenotype reminiscent of autophagy deficient mutants.

(C) Localization analysis of GFP-XopL of transgenic *A. thaliana* UBQ::GFP-XopL #23 line. Image represents single confocal planes from abaxial epidermal cells (bars = 5 µm).

(D) RT-qPCR analysis of *ATG8a* and *NBR1* transcript levels in *Arabidopsis thaliana* GFP or GFP-XopL plants. Values represent expression (n=3) relative to GFP control and were normalized to *PP2A*. Statistical significance (**P*<0.05) was revealed by Student's *t*-test.

Appendix Figure S8

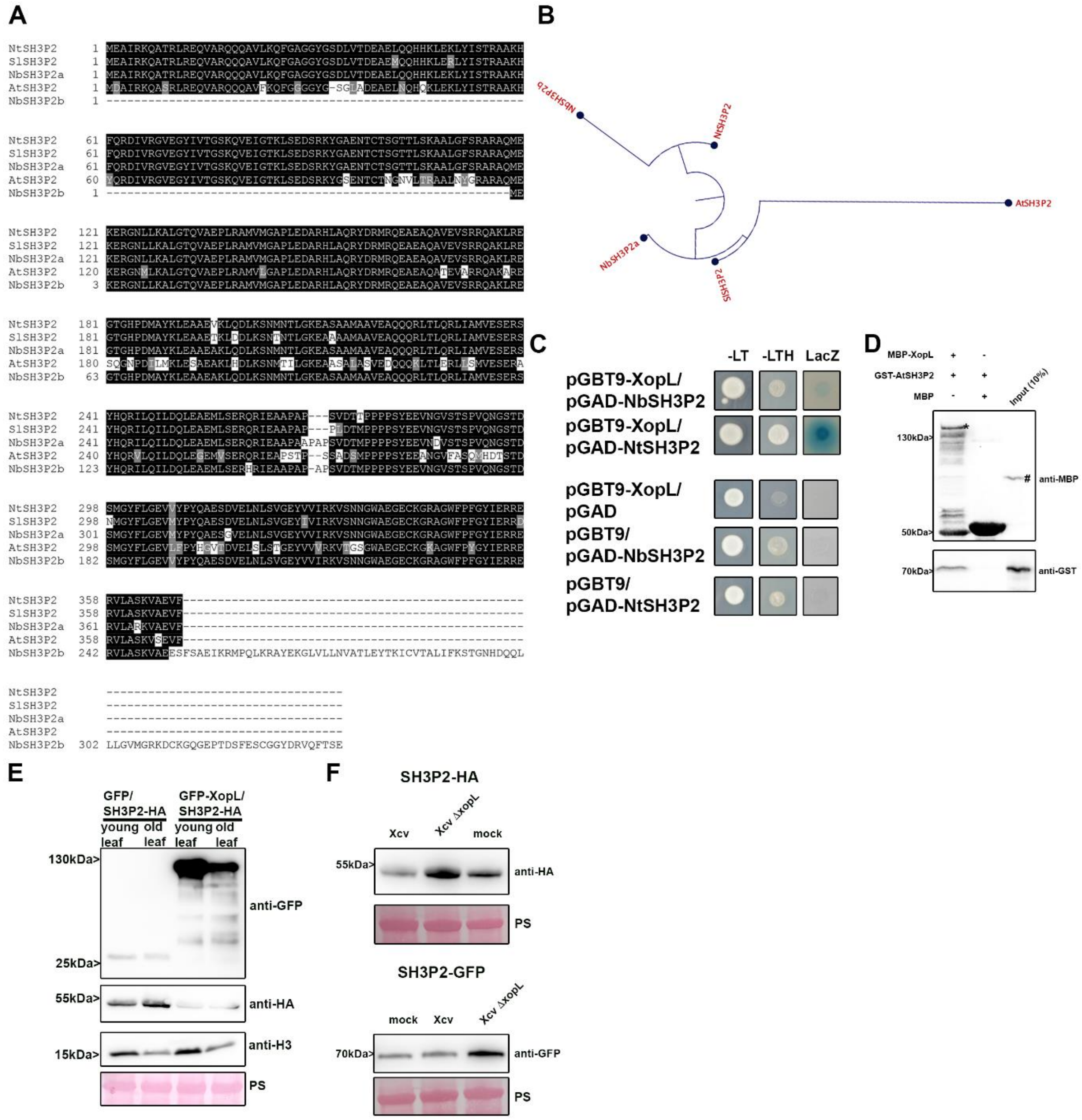

Fig. S8: SH3P2 is conserved in different plant species.

(A) Protein sequence alignment of SH3P2 from different species. The alignment was generated using CLUSTALW2 with default parameters and BoxShade 3.21. Positions of identical and similar sequences are boxed in black and grey, respectively. The following sequences were used to build the alignment: *Arabidopsis thaliana*, *Nicotiana tabacum, Nicotiana benthamiana*, *Solanum lycopersicum*.

(B) Phylogentic analysis of the SH3P2 from different plant species.

(C) XopL interacts with SH3P2 from *Nicotiana tabacum* and *Nicotiana benthamiana*. Interaction of XopL with SH3P2 in yeast two-hybrid assays. XopL fused to the GAL4 DNA-binding domain was expressed in combination with SH3P2 fused to the GAL4 activation domain (AD) in yeast strain Y190. Cells were grown on selective media before a LacZ filter assay was performed. The empty AD or BD vector served as negative control. NtSH3P2 = *Nicotiana tabacum* SH3P2, NbSH3P2 = *Nicotiana benthamiana* –LT = yeast growth on medium without Leu and Trp, –HLT = yeast growth on medium lacking His, Leu, and Trp, indicating expression of the HIS3 reporter gene. LacZ, activity of the lacZ reporter gene.

(D) *In vitro* co-IP assay showing direct interaction of XopL with AtSH3P2. MBP-XopL and GST-AtSH3P2 were expressed in E. coli. Pull down was performed using amylose resin. Proteins were detected in an immunoblot using antibodies as indicated. Asterisks indicate MBP-XopL full-length protein and hash indicates unspecific signal.

(E) XopL does not influence Histone H3 levels. Immunoblot analysis of protein levels in *N. benthamiana* plants expressing GFP/SH3P2-HA or GFP-XopL/SH3P2-HA. Expression of proteins were verified with indicated antibodies. Ponceau S staining serves as a loading control.

(F) SH3P2 protein levels during Xcv infection. *N. benthamiana roq1* plants were infiltrated with mock, Xcv or Xcv Δ*xopL* (oD_{600}=0.2) together with Agrobacteria expressing either 35S::SH3P2-HA or 35::SH3P2-GFP. Immunoblot was performed at 2dpi. Expression of SH3P2 was verified with indicated antibodies. Ponceau S staining serves as a loading control.

Movie EV1: XopL/SH3P2 puncta are mobile

Nicotiana benthamiana leaf epidermal cells transiently expressing $Venus^{N173}$-XopL in combination with AtSH3P2-$Venus^{C155}$.

Appendix Figure S9

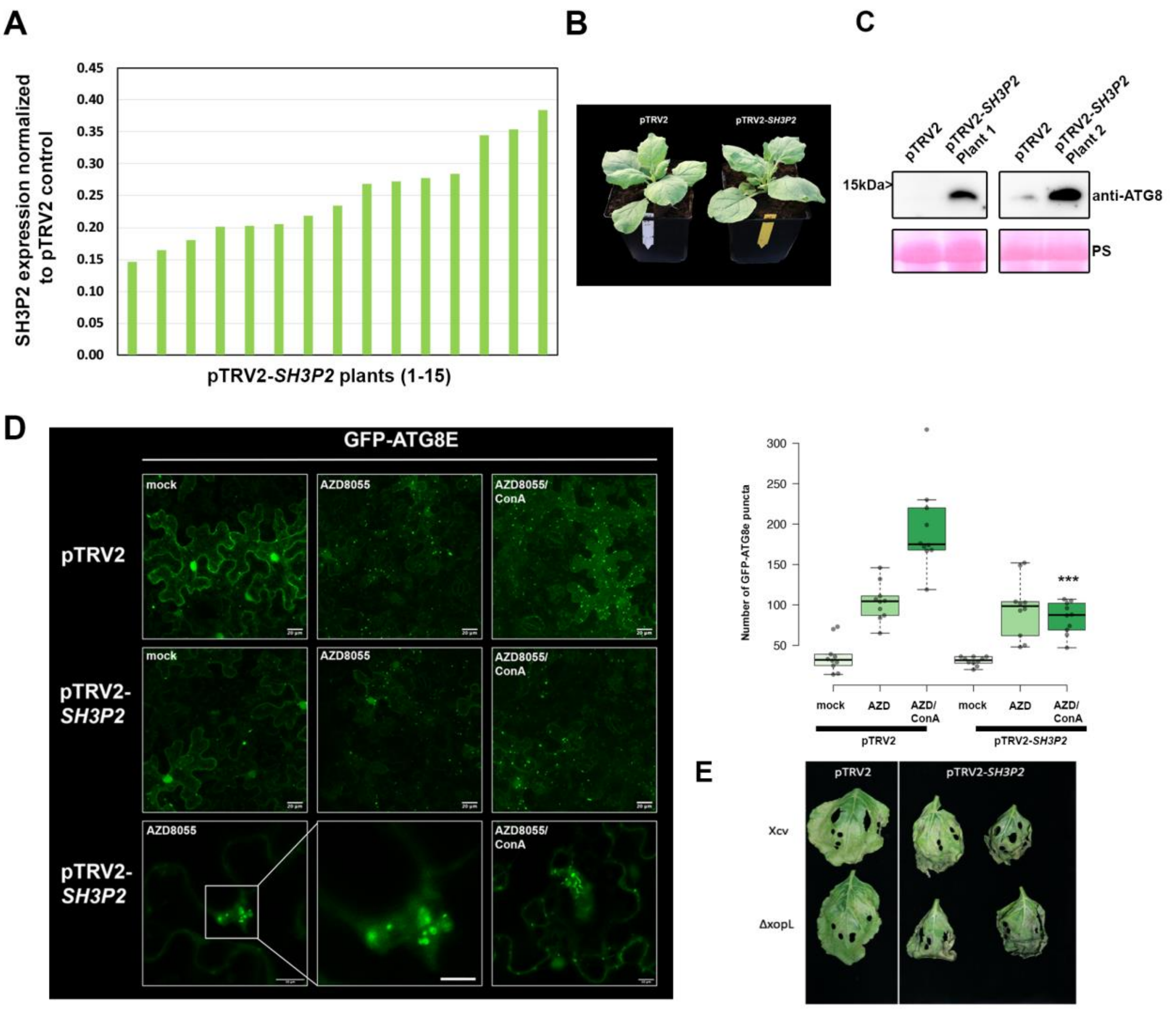

Fig. S9: Silencing of SH3P2 in *N. benthamiana* perturbs autophagy.

(A) qRT-PCR analysis of SH3P2 mRNA levels in silenced plants. *Actin* expression was used to normalize the expression value in each sample, and relative expression values were determined against pTRV2 control plants (set to 1).

(B) Phenotype of SH3P2-VIGS plants in comparison to the pTRV2 control. Picture was taken 14 dpi.

(C) Immunoblot analysis of ATG8 protein levels in *N. benthamiana* pTRV2 (control) and pTRV2-*SH3P2* (*SH3P2* silencing) 2 weeks after VIGS. Ponceau Staining (PS) served as a loading control. The experiment was repeated twice with similar results.

(D) GFP-ATG8e-labeled autophagosomes were quantified from pTRV2 or pTRV2-SH3P2 plants transiently expressing GFP-ATG8e at 2dpi in the presence of autophagy inducer AZD = AZD8055 and AZD/ConA. Puncta were calculated from z-stacks of n=10 individuals using ImageJ. Statistical significance (*** $P < 0.5$) was revealed by Student's t-test comparing number of autophagosomes in AZD/ConA treatments in pTRV2 and pTRV2-*SH3P2* plants. The experiment was repeated twice with similar results.

(E) Enhanced disease symptom development in pTRV2-*SH3P2* dip-inoculated plants with *Xcv* or *Xcv* Δ*xopL* at 10 dpi.

Appendix Figure S10

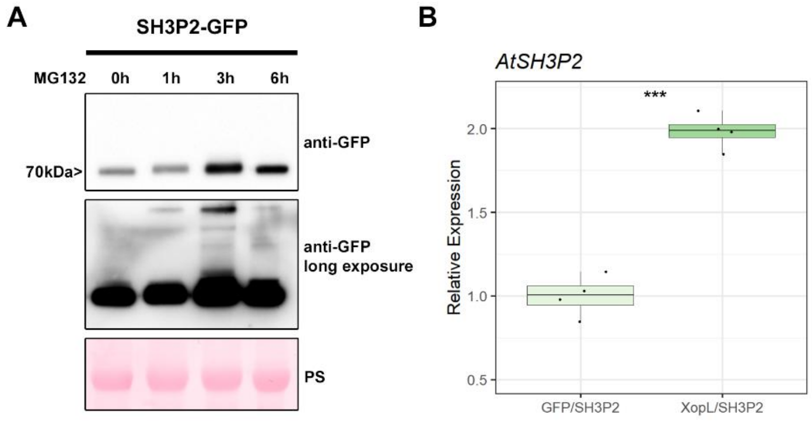

Fig. S10: Gene expression of SH3P2 is induced by XopL and XopL-mediated degradation is due to post-transcriptional degradation events.

(A) Immublot analysis of SH3P2-GFP protein after treatment with MG132 at 2dpi. Expression of SH3P2 was detected by anti-GFP antibody. Ponceau Staining (PS) served as a loading control.

(B) qRT-PCR analysis of AtSH3P2-specific mRNA levels in *N. benthamiana* plants transiently expressing AtSH3P2-HA, upon coexpression with GFP or GFP-XopL. *Actin* expression was used to normalize the expression value in each sample. Values represent *AtSH3P2* transcript level normalized to control (n=4).

Appendix Figure S11

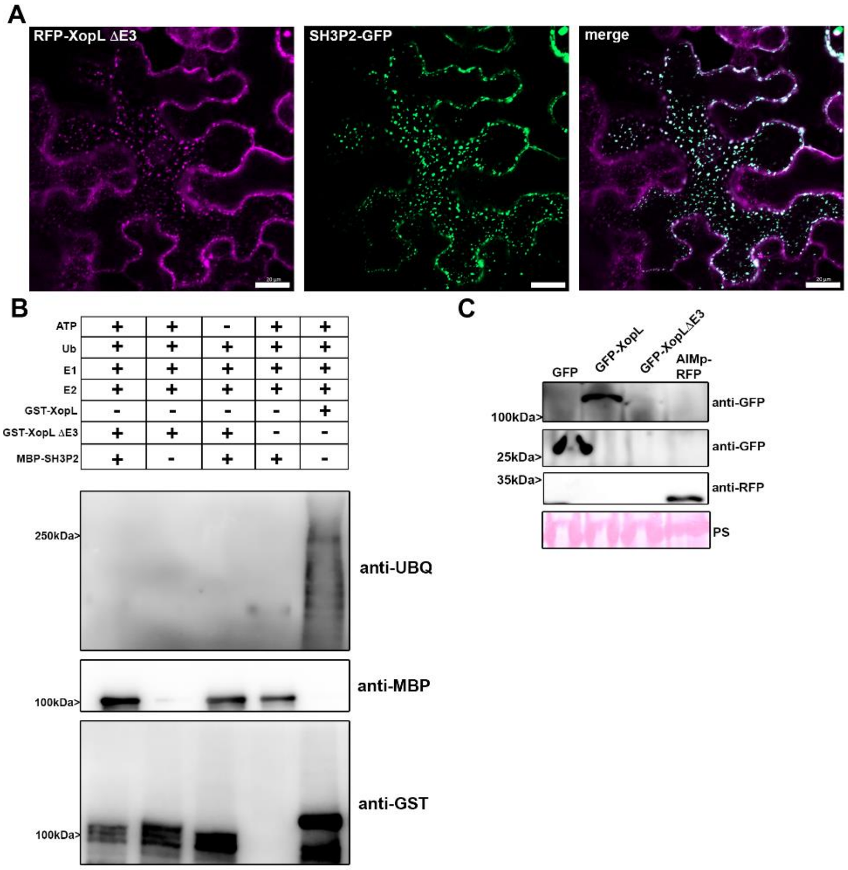

Fig. S11: RFP-XopL ΔE3 co-localizes with and is unable to ubiquitinate SH3P2-GFP.

(A) Colocalization analysis of RFP-XopL ΔE3 with SH3P2-GFP in *N. benthamiana* leaves. Imaging was performed 2 d after transient expression and images represent single confocal planes from abaxial epidermal cells (bars = 20 μm).
(B) *In vitro* ubiquitination assay reveals GST-XopL ΔE3 is unable to ubiquitinate MBP-SH3P2. GST-XopL, ΔE3 and MBP-SH3P2 were tested using the Arabidopsis His-AtUBA1 and His-AtUBC8. Lanes 2 to 4 are negative controls, while Lane 5 is the positive control with GST-XopL. Proteins were separated by SDS-PAGE and detected by immunoblotting using the indicated antibodies. The experiment was repeated three times with similar results.
(C) Expression control for quantitative luciferase assay in Figure 4E: GFP, GFP-XopL, GFP-XopL ΔE3 and RFP-AIMp were expressed together with luciferase constructs in *N. benthamiana*. Samples were taken at 2 dpi, total proteins extracted and immunoblotted using the indicated antibodies. Ponceau Staining (PS) served as a loading control.

Appendix Figure S12

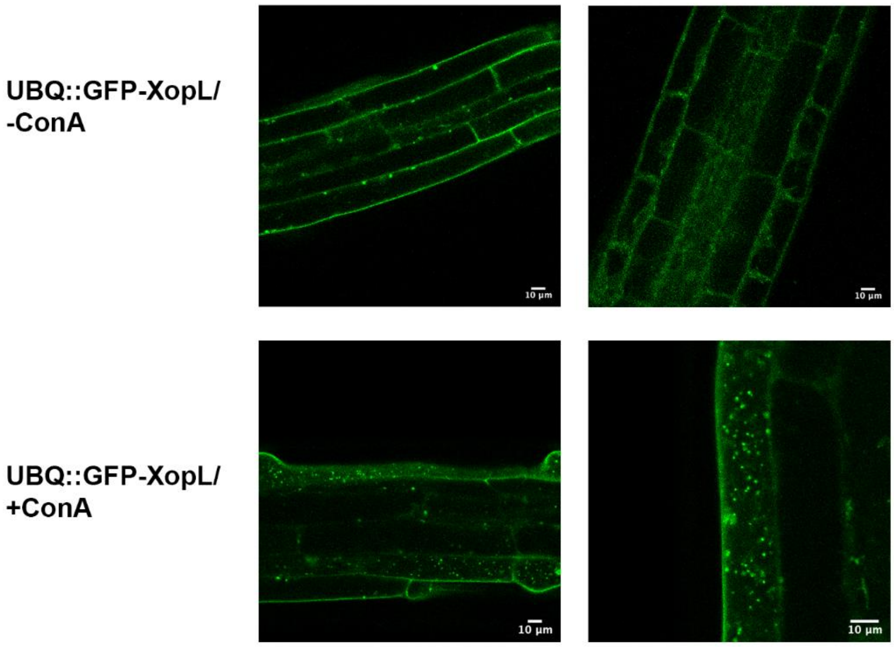

Fig. S12: XopL is degraded in the vacuole. Localization of GFP-XopL in the presence or absence of ConA in transgenic GFP-XopL. DMSO or 0.5 μM ConA was used to treat seedlings, followed by confocal imaging of the roots. GFP-labeled puncta detectable upon ConA treatment indicate XopL accumulation in the vacuole (bars = 20 μm).

Appendix Figure S13

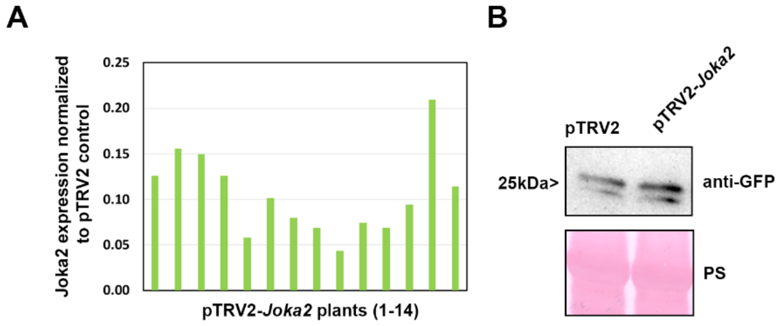

Fig. S13: Virus-induced gene silencing of *Joka2* in *N. benthamiana* plants.
(A) qRT-PCR analysis of Joka2 mRNA levels in *Joka2* silenced pepper plants. *Actin* expression was used to normalize the expression value in each sample, and relative expression values were determined against pTRV2 control plants (set to 1)**.**
(B) Immunoblot analysis of GFP and GFP-XopL protein levels in *N. benthamiana* plants silenced for *Joka2* (pTRV2-Joka2) compared against control (pTRV2). Ponceau staining (PS) served as a loading control.

Appendix Figure S14

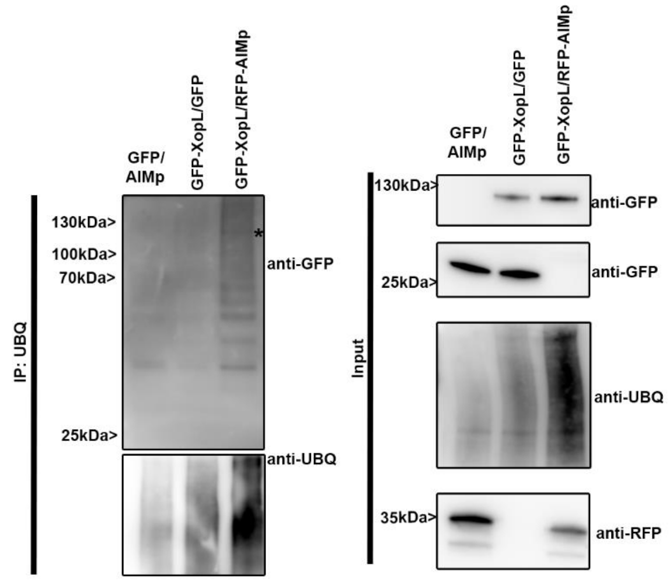

Fig. S14: XopL in planta ubiquitination is enhanced by the presence of AIMp GFP-XopL or GFP were transiently expressed in *N. benthamiana*. RFP-AIMp was co-infiltrated. Samples were taken 48 hpi, and total proteins (Input) were subjected to immunoprecipitation (IP) with the ubiquitin pan selector, followed by immunoblot analysis of the precipitates using either anti-GFP or anti-ubiquitin antibodies. RFP-AIMp expression was verified by an anti-RFP antibody. Asterisk indicates the GFP-XopL full-length protein. The experiment was repeated twice with similar results.

Appendix Figure S15

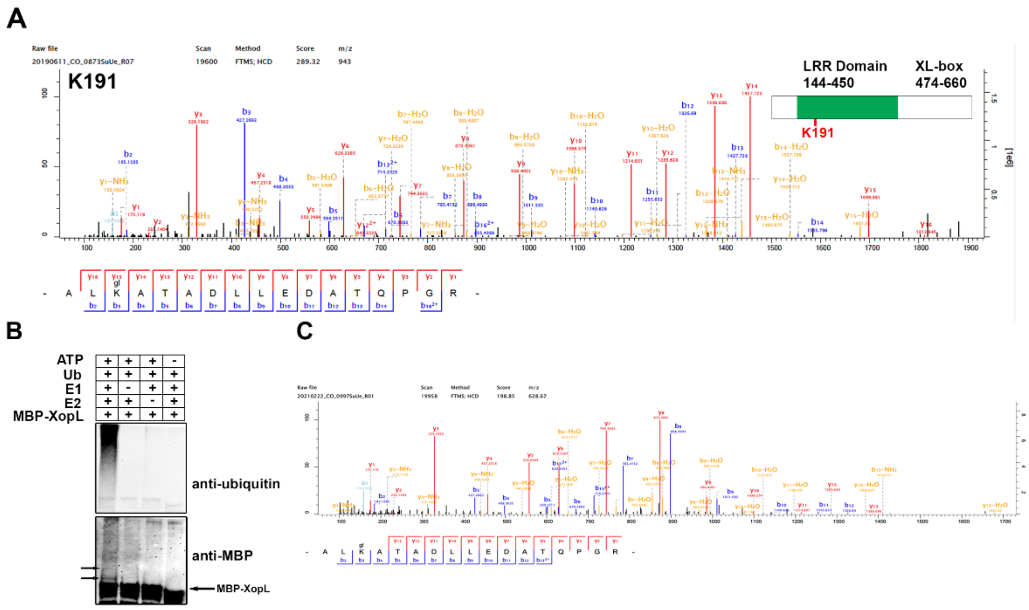

Fig. S15: XopL is ubiquitinated *in planta* and undergoes self-ubiquitination
(A) XopL ubiquitination site at lysine 191 was identified in vivo by LC-MS/MS. GFP-XopL was transiently expressed in *N. benthamiana* and total proteins were subjected to anti-GFP IP followed by trypsin digestion. Ubiquitinated peptides were detected by LC-MS/MS. The spectrum shows the fragmentation pattern of the GlyGly modified peptide ALglKATADLLEDATQPGR corresponding to amino acids 189-205.
(B) *In vitro* ubiquitination assay reveals autoubiquitination of XopL. Ubiquitination of MBP-XopL was tested using the Arabidopsis His-AtUBA1 and His-AtUBC8. Lanes 2 to 4 are negative controls. Proteins were separated by SDS-PAGE and detected by immunoblotting using the indicated antibodies. Arrows in the MBP blot indicate higher molecular weight bands of MBP-XopL and autoubiquitination events. The experiment was repeated twice with similar results.
(C) XopL ubiquitination site at lysine 191 was identified in vitro by LC-MS/MS. GST-XopL was used in an in vitro ubiquitination assay and samples were subjected to trypsin digestion. Ubiquitinated peptides were detected by LC-MS/MS. The spectrum shows the fragmentation pattern of the GlyGly modified peptide ALglKATADLLEDATQPGR corresponding to amino acids 189-205.

Appendix Figure S16

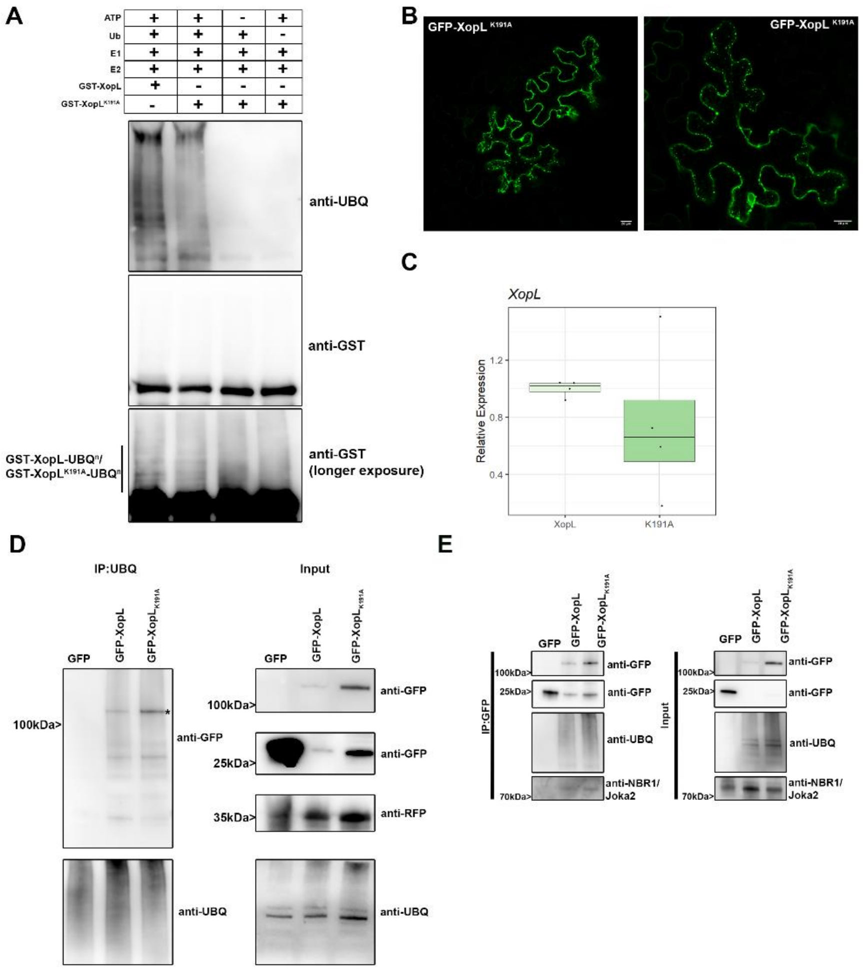

Fig. S16: Characterization of XopL K191A variant in vitro and in planta.

(A) *In vitro* ubiquitination assay reveals less autoubiquitination of XopL K191A compared to XopL WT. Ubiquitination of GST-XopL was tested using the Arabidopsis His-AtUBA1 and His-AtUBC8. Lanes 3 to 5 are negative controls. Proteins were separated by SDS-PAGE and detected by immunoblotting using the indicated antibodies. The experiment was repeated twice with similar results.

(B) Localization analysis of GFP-XopL$_{K191A}$ in *N. benthamiana* leaves. Imaging was performed 2 d after transient expression and images represent single confocal planes from abaxial epidermal cells (bars = 20 μm).

(C) qRT-PCR analysis of GFP-XopL and GFP-XopL K191A mRNA levels in *N. benthamiana* plants during transient expression. *Actin* expression was used to normalize the expression value in each sample.

(D) GFP-XopL and GFP-XopL$_{K191A}$ were transiently expressed in *N. benthamiana*. RFP-AIMp was co-infiltrated. Samples were taken 48 hpi, and total proteins (Input) were subjected to immunoprecipitation (IP) with the ubiquitin pan selector, followed by immunoblot analysis of the precipitates using either anti-GFP or anti-ubiquitin antibodies. GFP served as a control. RFP-AIMp expression was verified by an anti-RFP antibody. Asterisk indicates the GFP-XopL full-length protein. The experiment was repeated twice with similar results.

(E) GFP-XopL and GFP-XopL$_{K191A}$ were transiently expressed in *N. benthamiana*. Samples were taken 48 hpi, and total proteins (Input) were subjected to immunoprecipitation (IP) with GFP-Trap beads, followed by immunoblot analysis of the precipitates using either anti-GFP,

anti-ubiquitin and anti-NBR1 antibodies. GFP served as a control. Asterisk indicates the GFP-XopL full-length protein. The experiment was repeated three times with similar results.

Appendix Figure S17

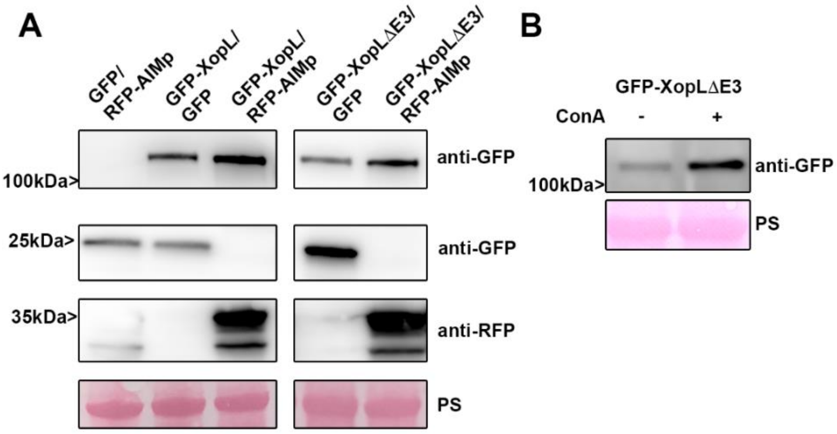

Fig. S17: XopL ΔE3 is degraded by autophagy.
(A) GFP, GFP-XopL, or GFP-XopL ΔE3 was coexpressed with RFP-AIMp or RFP control in *N. benthamiana*. Samples were taken at 2 dpi, total proteins extracted and immunoblotted using the indicated antibodies. Ponceau Staining (PS) served as a loading control.
(B) Immunoblot of transiently expressed GFP-XopL ΔE3 in *N. benthamiana* after treatment of ConA or DMSO carrier. Ponceau Staining (PS) served as a loading control.

Appendix Figure S18

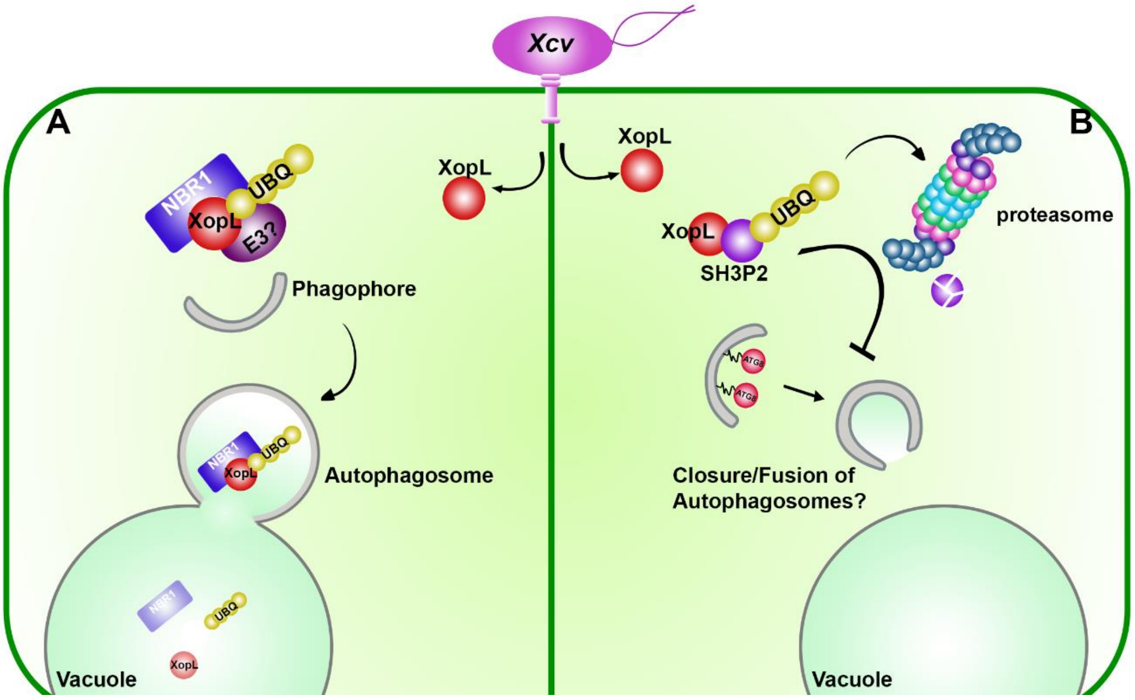

Fig. S18: Model illustrating the function of XopL
(A) Xenophagy of XopL: Upon delivery of XopL in the plant XopL undergoes self-ubiquitination and possible ubiquitination by an unknown host E3 ligase. Joka2/NBR1

associates with XopL and triggers its degradation via the selective autophagy pathway in the vacuole. **(B) XopL blocks autophagy**: XopL interacts with autophagy component SH3P2 inside the cell and ubiquitinates it to degrade it via the 26S proteasome. Degradation of SH3P2 results in defects of autophagosome delivery into the vacuole and hence suppresses autophagy.

Appendix Figure S19

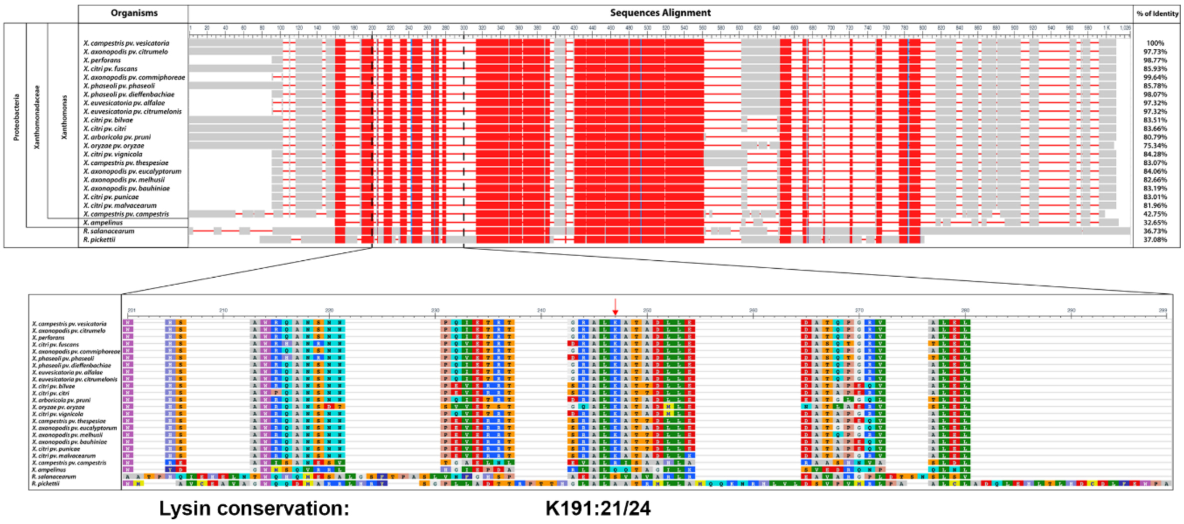

Fig. S19: XopL K191 residue is highly conserved through the Xanthomonas genus
(A) Sequence alignment of XopL protein from *Xcv* with related effectors from *Xanthomonas* genus or more distantly related plant pathogen bacteria. Colors represent amino acid conservation through the alignment, with red for highly conserved residues, blue for lower conservation and grey for no conservation. Identical amino acid percentage to XopL^{Xcv} is displayed to the right of the alignment.
(B) Closer view of the region 201-299 of the alignment with amino acids colored according to the Rasmol coloration. Lysine K191 is indicated with a red arrow.

Appendix Table S1

Method/Name	Sequence
**qPCR**	
NbActin Fwd	5'-AAAGACCAGCTCATCCGTGGAGAA-3'
NbActin Rev	5'-TGTGGTTTCATGAATGCCAGCAGC-3'
NbATG8-2.1/2 Fwd	5'-CACCCACTTGAAAGGCGACAGGC-3'
NbATG8-2.1/2 Rev	5'-GCCTTCTCAGCACTAAGCTTTATTCTC-3'
NbATG8-1.1/2 Fwd	5'-CTTGAGAAGAGGCGTGCTGAAGC-3'
NbATG8-1.1/2 Rev	5'-ATCTGTTGGTGGTAGGACATTATCAAC-3'
NbJoka2 Fwd	5'-CGTTGTGATGGTTGTGGTGT-3'
NbJoka2 Rev	5'-AGGGACGCCGGTAAGTTAAA-3'
NbSH3P2 Fwd	5'-TACTATGCCTCCACCTCCCT-3'
NbSH3P2 Rev	5'-TTTGCATTCACCTTCAGCCC-3'
NbJoka2 Fwd	5'-CGCAGCTGGATCTGACATTA-3'
NbJoka2 Rev	5'-CGGGAGCAGAAGAACTTGAC-3'
NbATG7 Fwd	5'-ATGGCGGATAGTGGAAGAGG-3'
NbATG7 Rev	5'-CCTGACTAGCTAGCCGAGAC-3'
NBR1 Fwd	5'-GAGGACCCAGACCGGAAGG-3'
NBR1 Rev	5'-GACAAACACGACGAGGATGC-3'
ATG8a Fwd	5'-CAAGCTTGGAGCTGAGAAAG-3'
ATG8a Rev	5'-GCAACGGTAAGAGATCCAAA-3'
**Cloning**	
NbSH3P2-VIGS_cloning_Fwd	5'-CTAGCTCAAAGATATGATAGAATGCGA-3'
NbSH3P2-VIGS_cloning_Rev	5'-GTGGTAGCTGATCAAGAATCTGAAGGAT-3'
XopL Fw	5'-CACCATGCGACGCGTCGATCAAC-3'
XopL Rev	5'-CTACTGATGGCCTGAAGGTTCCGG-3'
AtSH3P2 Fwd (with STOP)	5'-CACCATGGATGCAATTAGAAAACAAGC-3'
AtSH3P2 Rev	5'-TCAGAAAACTTCGGACACTTTGCTAGC-3'
AtSH3P2 Fwd (wihout STOP)	5'-CACCAACAATGGATGCAATTAGAAAACAAGC-3'
AtSH3P2 Rev	5'-GAAAACTTCGGACACTTTGCTAGCAAG-3'
NbSH3P2 Fwd (with STOP)	5'-GAATTCATGGAAGCAATCAGAAAGCAAGC-3'
NbSH3P2 Rev	5'-GTCGACTCAGAAAACCTCGGCAACTTTCC-3'
SlJoka2 Fwd	5'-GGGGACAAGTTTGTACAAAAAAGCAGGCTTC ATGTGTGAGTTGGGGCTAT-3'
SlJoka2 Rev	5'-GGGGACCACTTTGTACAAGAAAGCTGGGTTCTACTGCTCTCCAGCAATAA-3'
**Site-directed Mutagenesis**	
XopL K191A sense	5'-CGCACAGGCCGGGCGCTGGCGGCGACAGCCGACCTGCTGGA-3'
XopL K191A antisense	5'-CTCCAGCAGGTCGGCTGTCGCCGCCAGCGCCCGGCCTGTGCG-3'
XopLH584A L585A G586E sense	5'-CAGCACATGACGATCGACGCGGCAGGGTTGATGGAGCTCCCGGA-3'
XopLH584A L585A G586E antisense	5'-TCCGGGAGCTCCATCAACCCTGCCGCGTCGATCGTCATGTGCTG-3'
**Construction of null mutants and complementation in Xcv**	
XopQ up Fwd	5'-GCCCTTTCGTCTTCAAGGCCGCCTGCGCCTGCTCA-3'
XopQ up Rev	5'-CGGAGCGCGGGAGGACCTCTGGCAGTGAAAG-3'
XopQ down Fwd	5'-TCCTCCCGCGCTCCGCAGCTTTCGC-3'
XopQ down Rev	5'-AAGCTGTCAAACATGAGGCTGTATCCGGCCGGTTG-3'
XopL up Fwd	5'-GCCCTTTCGTCTTCAAGGCGGATTGACTGCGGTTG-3'
XopL up Rev	5'-TGGCTCTCGCATTCCTCGTCTTGGC-3'
XopL down Fwd	5'-GGAATGCGAGAGCCAACGCGACAGGC-3'
XopQ down Rev	5'-AAGCTGTCAAACATGAGTGCCGCCAATCAGGAAGC-3'
XopL comp Fwd	5'-GGCACGACAGGTTTGATGGCAACGCGTGCTGACGC-3'
XopL comp Rev	5'-TGGTGATGATGGTGGATCTGATGGCCTGAAGGTTCCG-3'
