# Supplementary figures and images for: A bacterial effector counteracts host autophagy by promoting degradation of an autophagy component
Source: EMBO J. 2022 May 27;41(13):e110352. doi: 10.15252/embj.2021110352 (PMC9251887; doi:10.15252/embj.2021110352)

Figure 1A

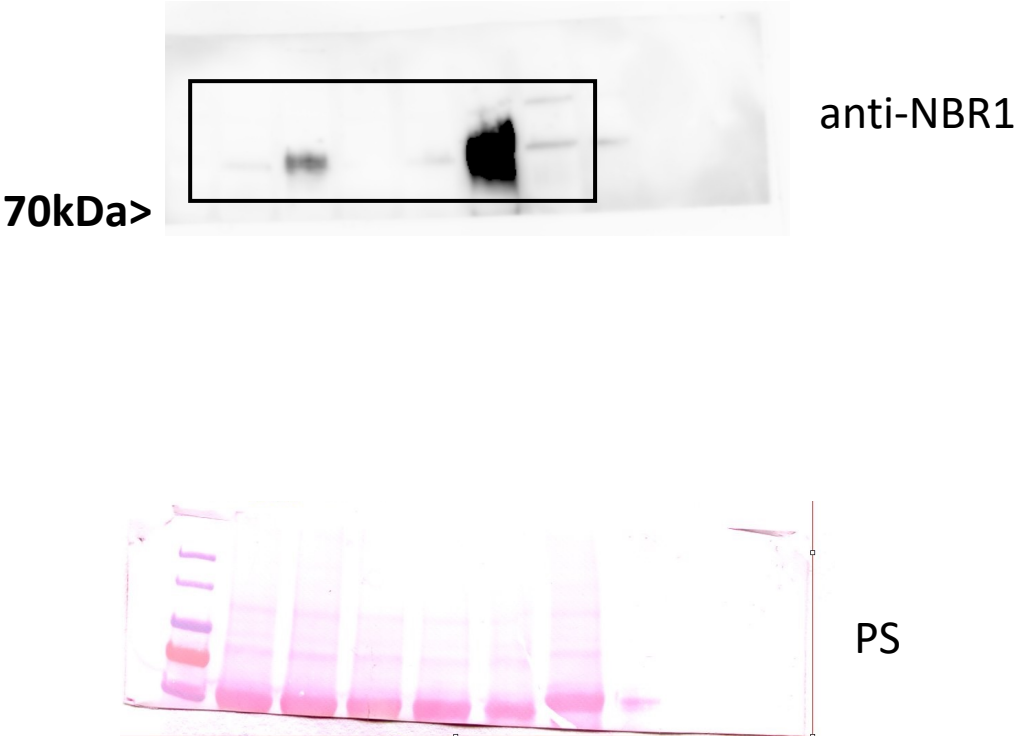

Figure 1B

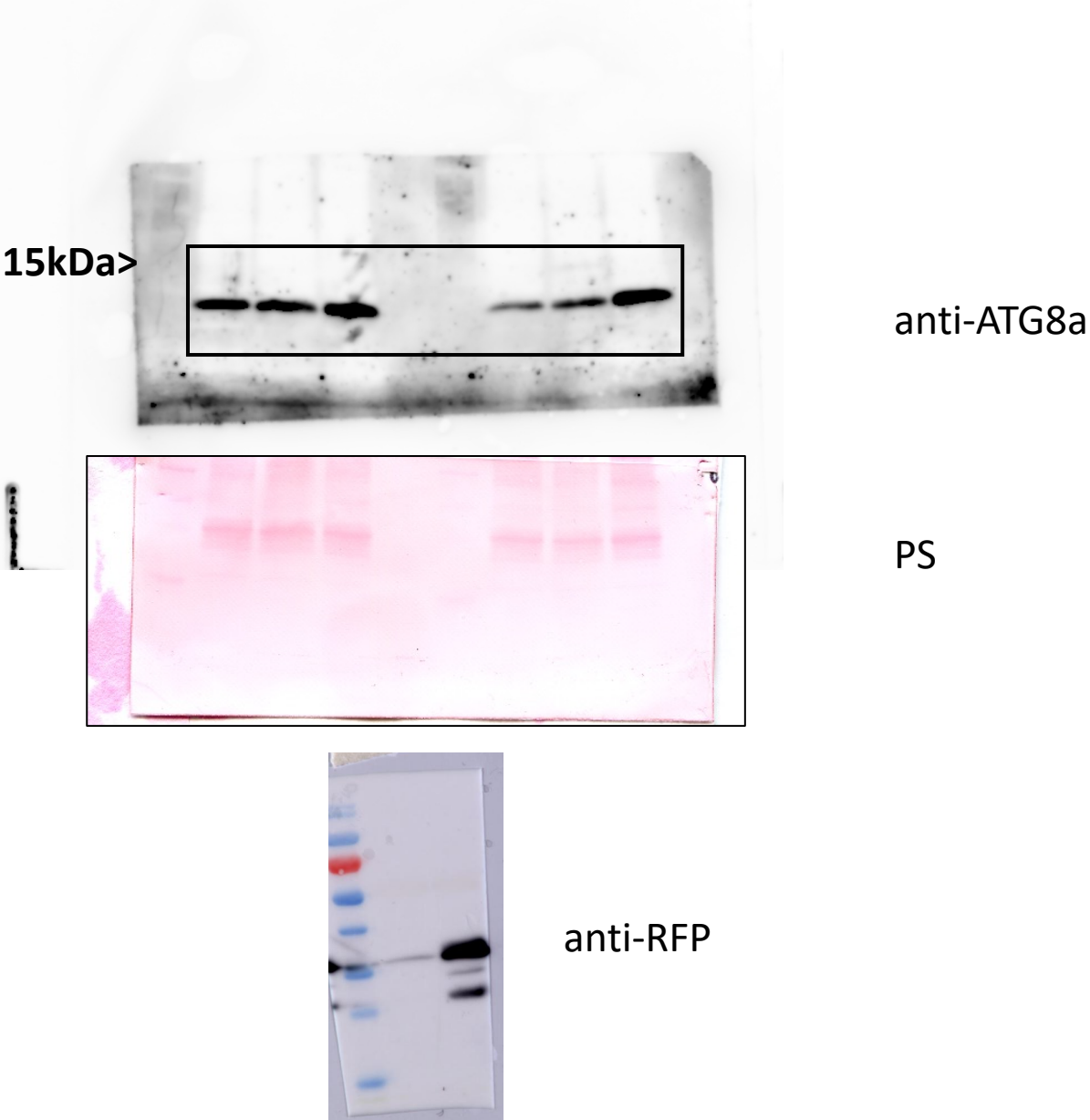

Supplement: Supplementary file 3 — Source Data for Figure 1 [file EMBJ-41-e110352-s003.pdf]

Figure 2B

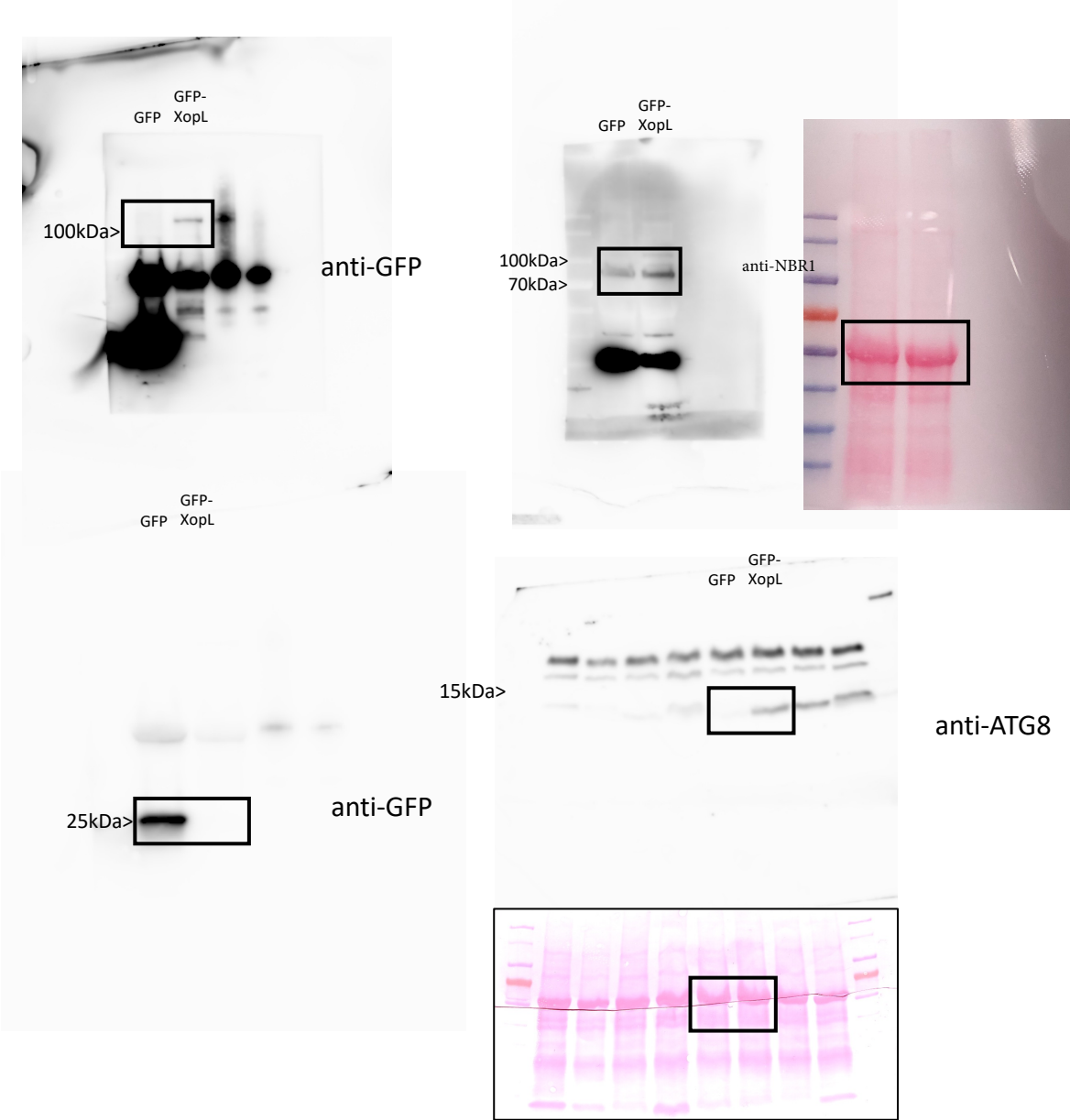

Figure 2C

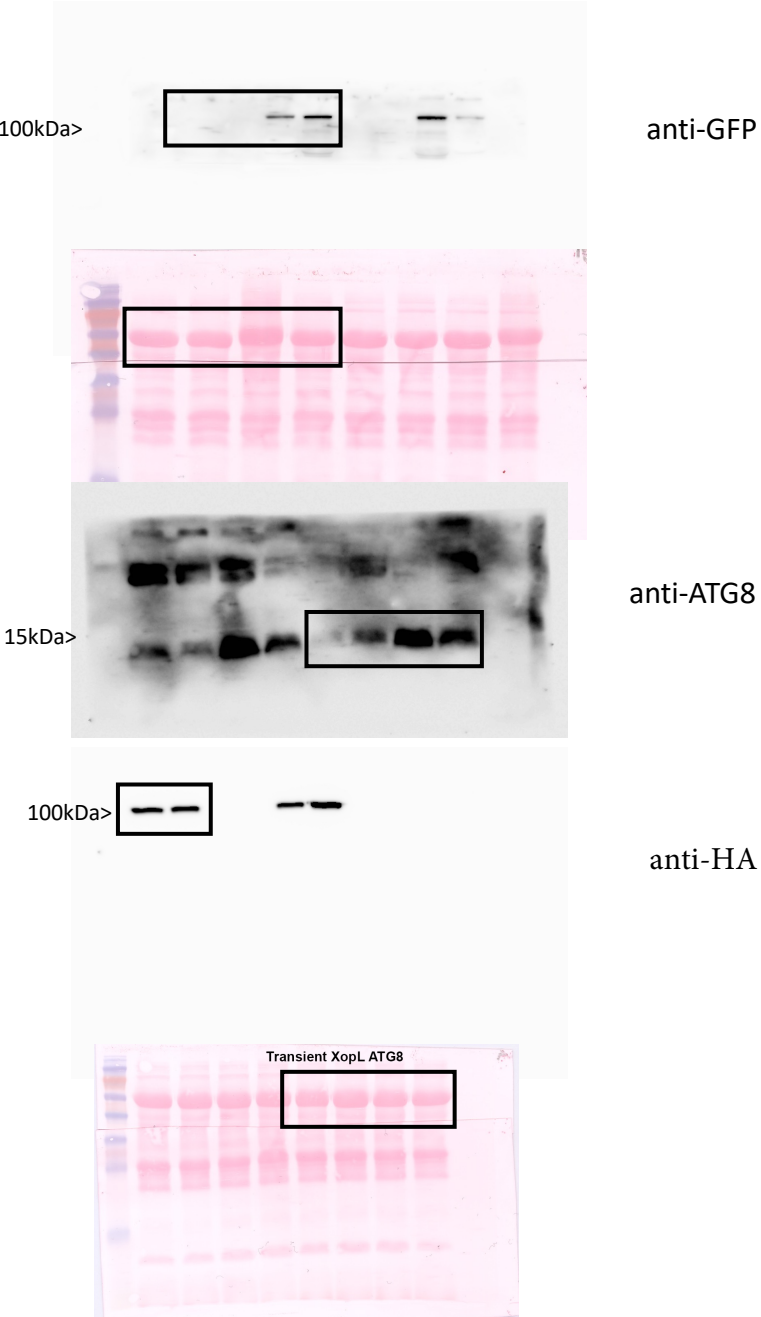

**Figure 2E**

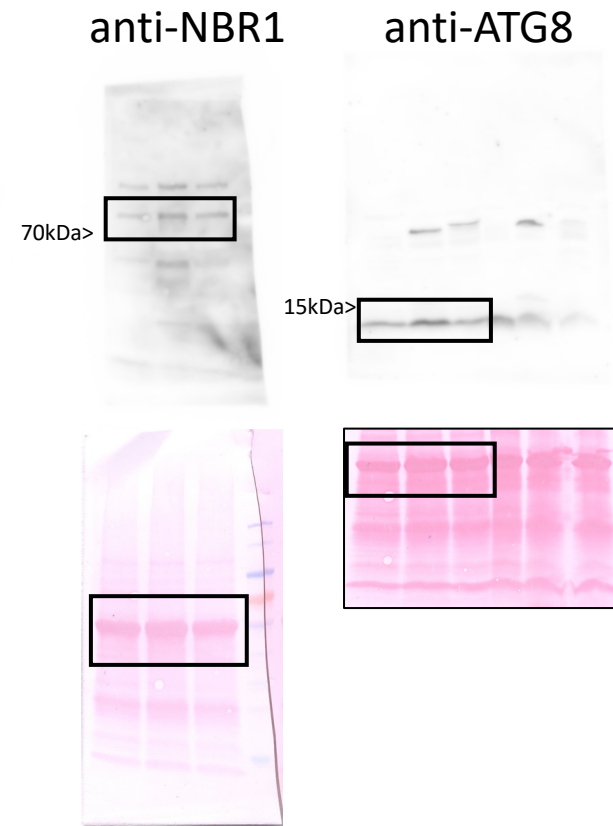

Supplement: Supplementary file 4 — Source Data for Figure 2 [file EMBJ-41-e110352-s008.pdf]

Figure 3B

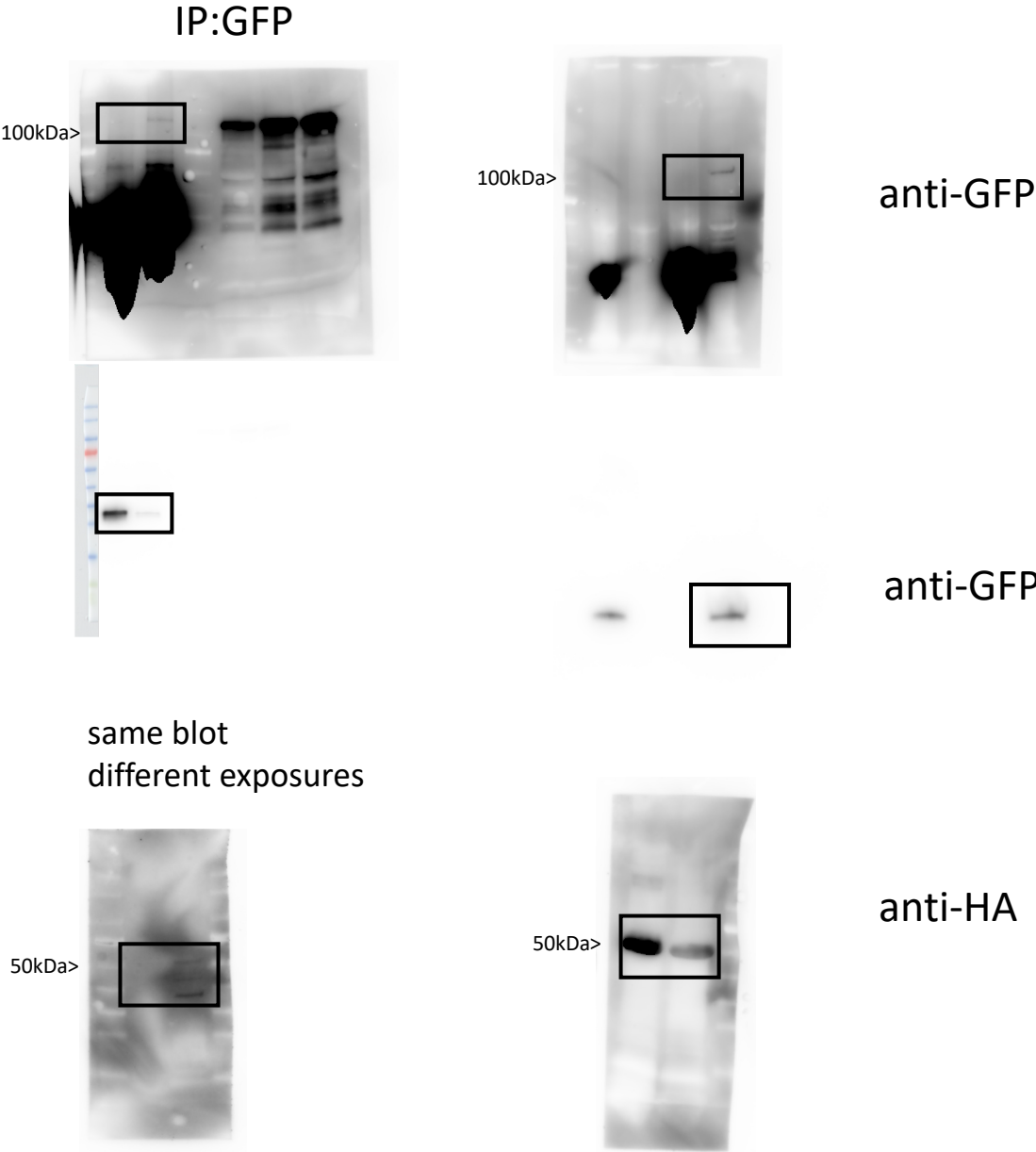

Figure 3E

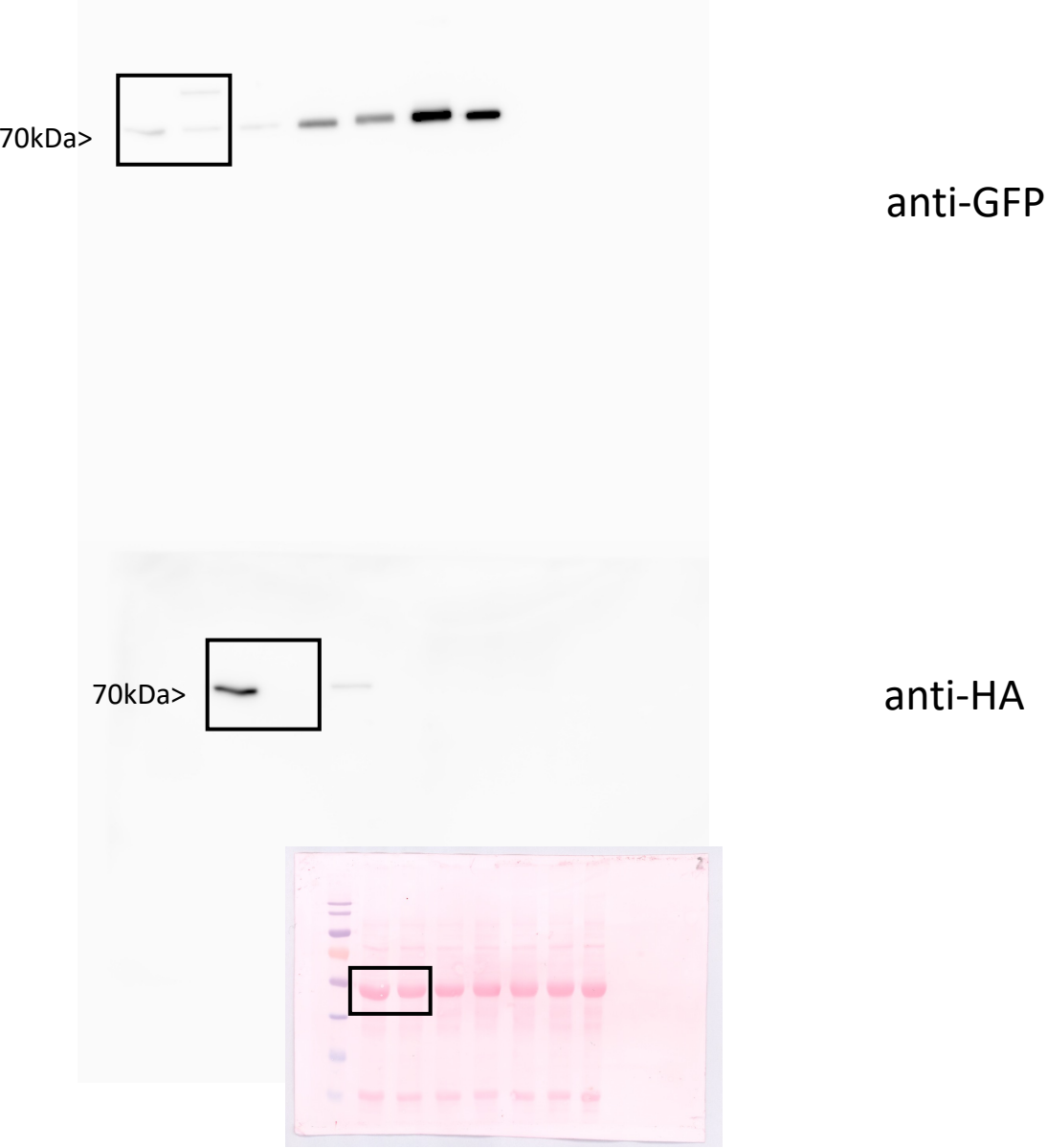

Supplement: Supplementary file 5 — Source Data for Figure 3 [file EMBJ-41-e110352-s002.pdf]

Figure 4A

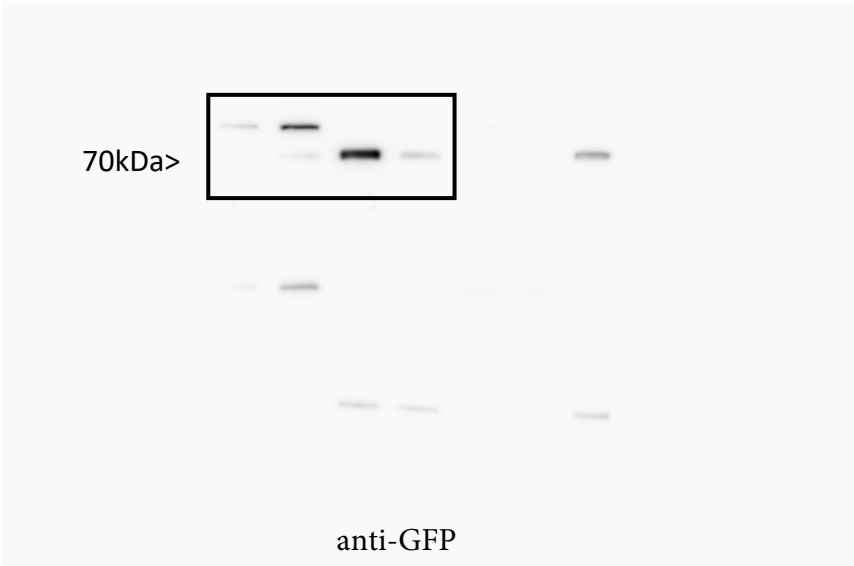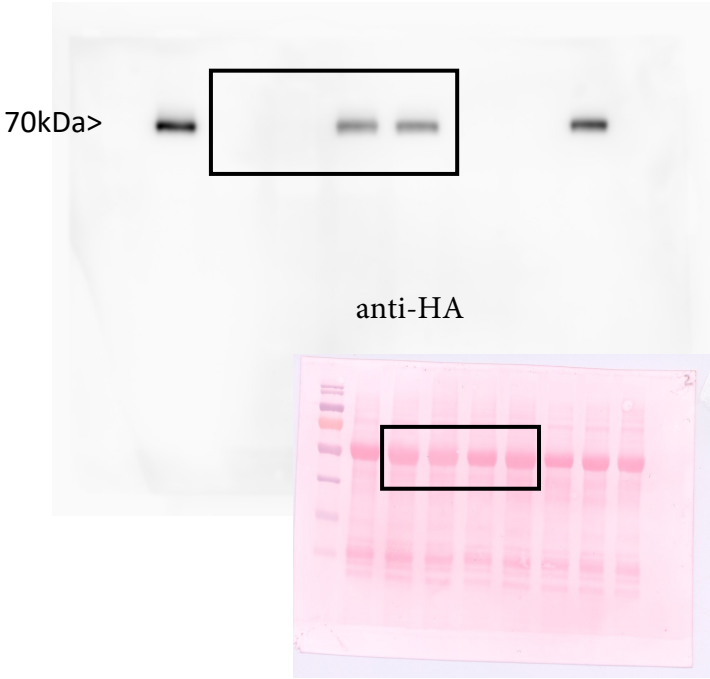

Figure 4B

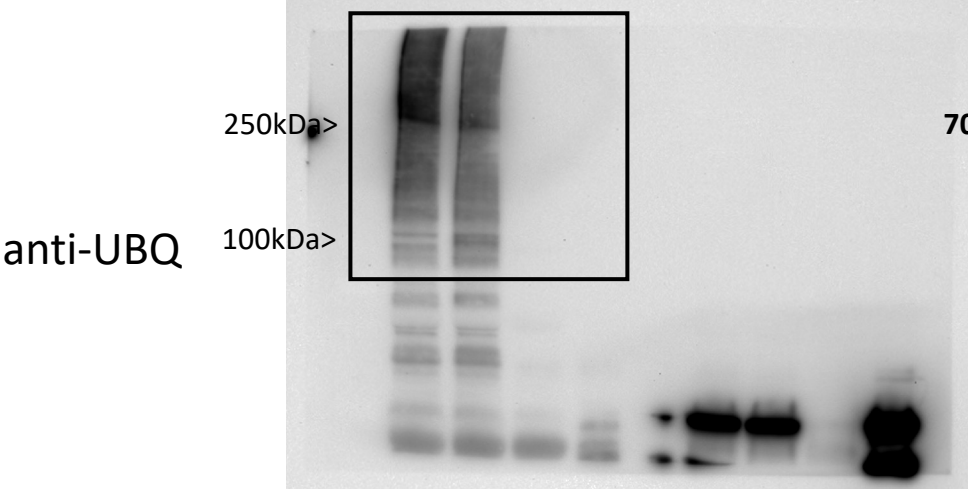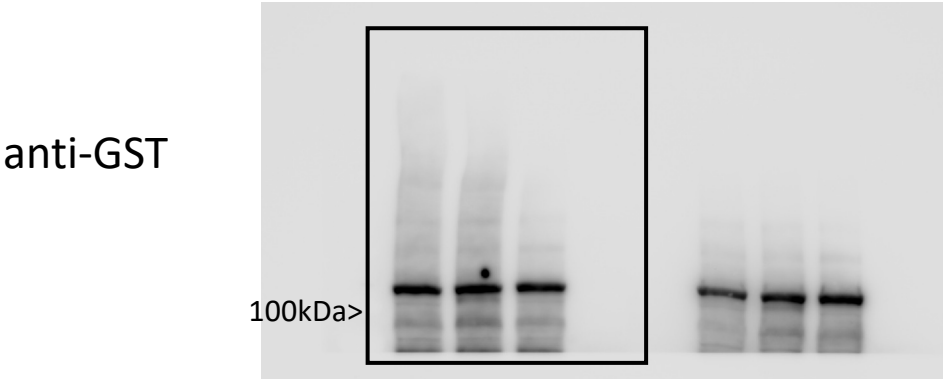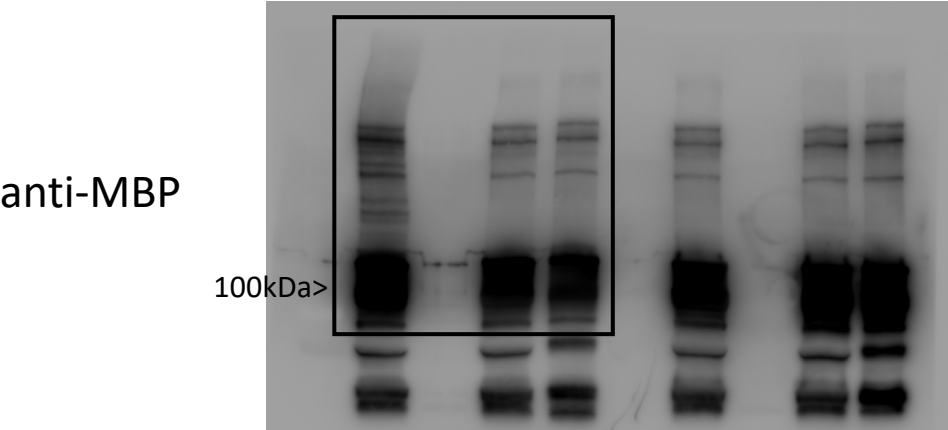

4C

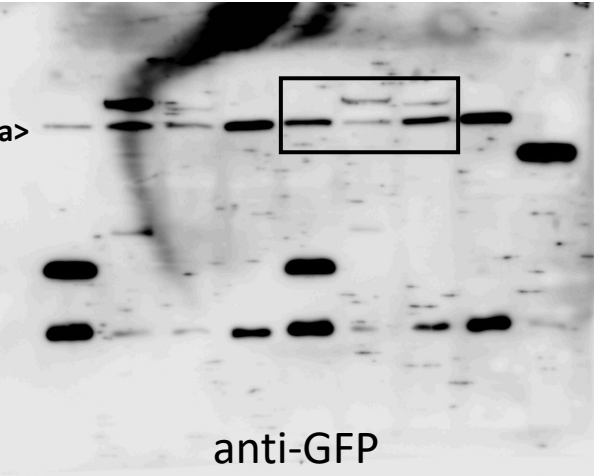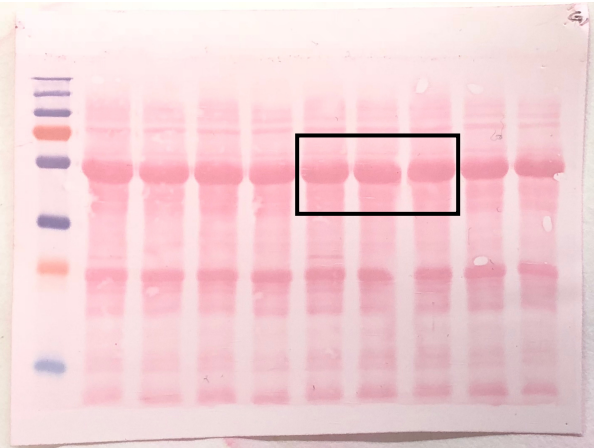

4D

100kDa>

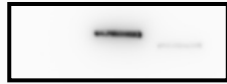

anti-GFP

25kDa>

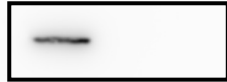

15kDa>

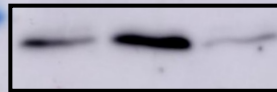

anti-ATG8

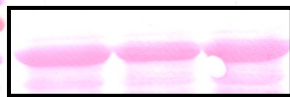

Supplement: Supplementary file 6 — Source Data for Figure 4 [file EMBJ-41-e110352-s004.pdf]
