# Supplementary material for: A bacterial effector counteracts host autophagy by promoting degradation of an autophagy component
Source: EMBO J. 2022 May 27;41(13):e110352. doi: 10.15252/embj.2021110352 (PMC9251887; doi:10.15252/embj.2021110352)
Supplement: Supplementary file 7 — Source Data for Figure 5 [file EMBJ-41-e110352-s007.pdf]

5A

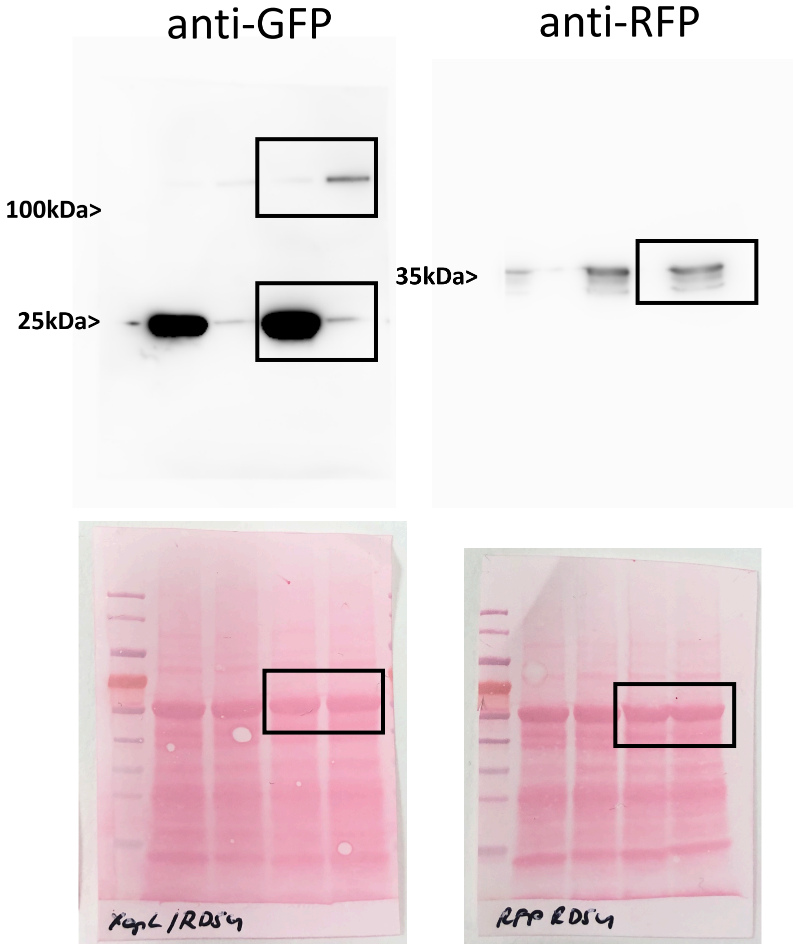
anti-GFP
anti-RFP
100kDa>
35kDa>
25kDa>

5D

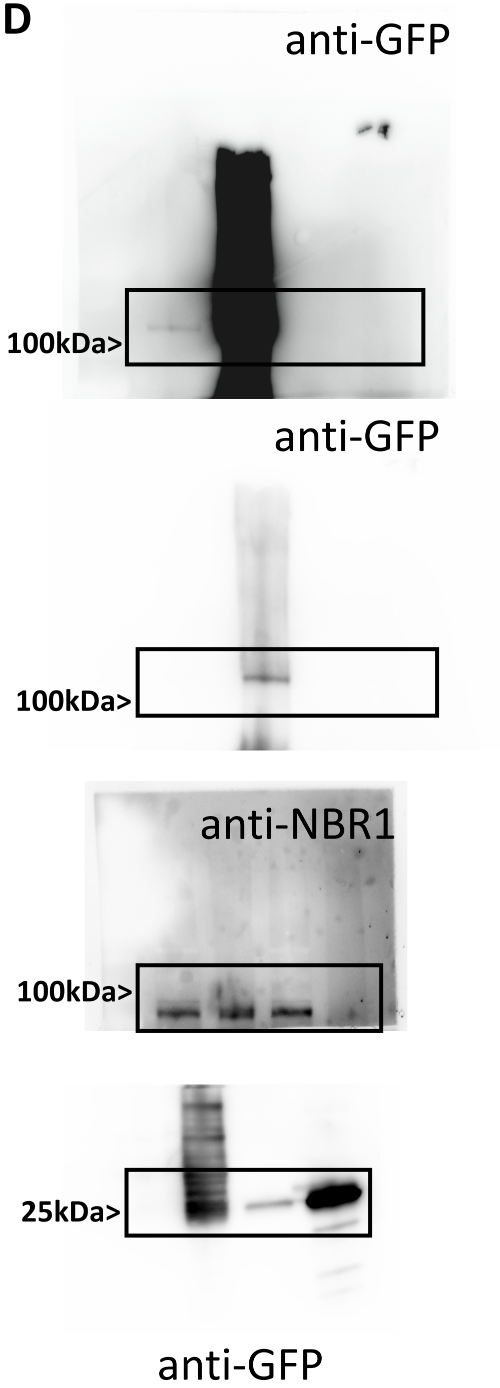
anti-GFP
100kDa>
anti-GFP
100kDa>
anti-NBR1
100kDa>
25kDa>
anti-GFP

5E

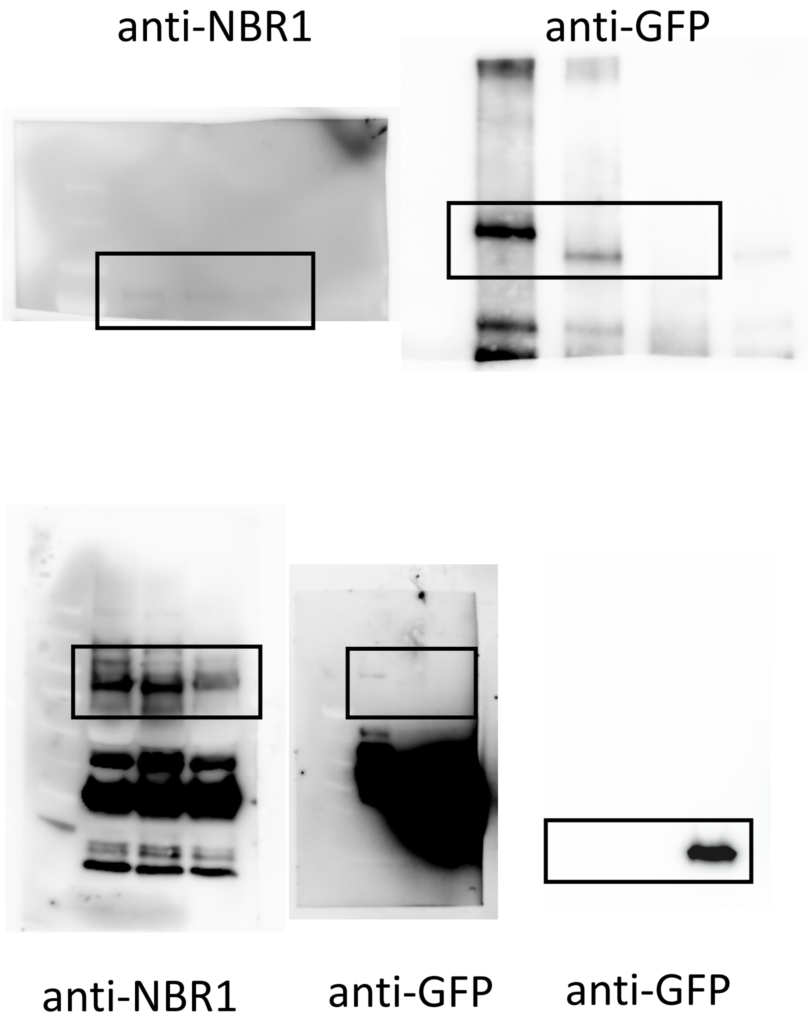
anti-NBR1
anti-GFP
anti-NBR1
anti-GFP
anti-GFP

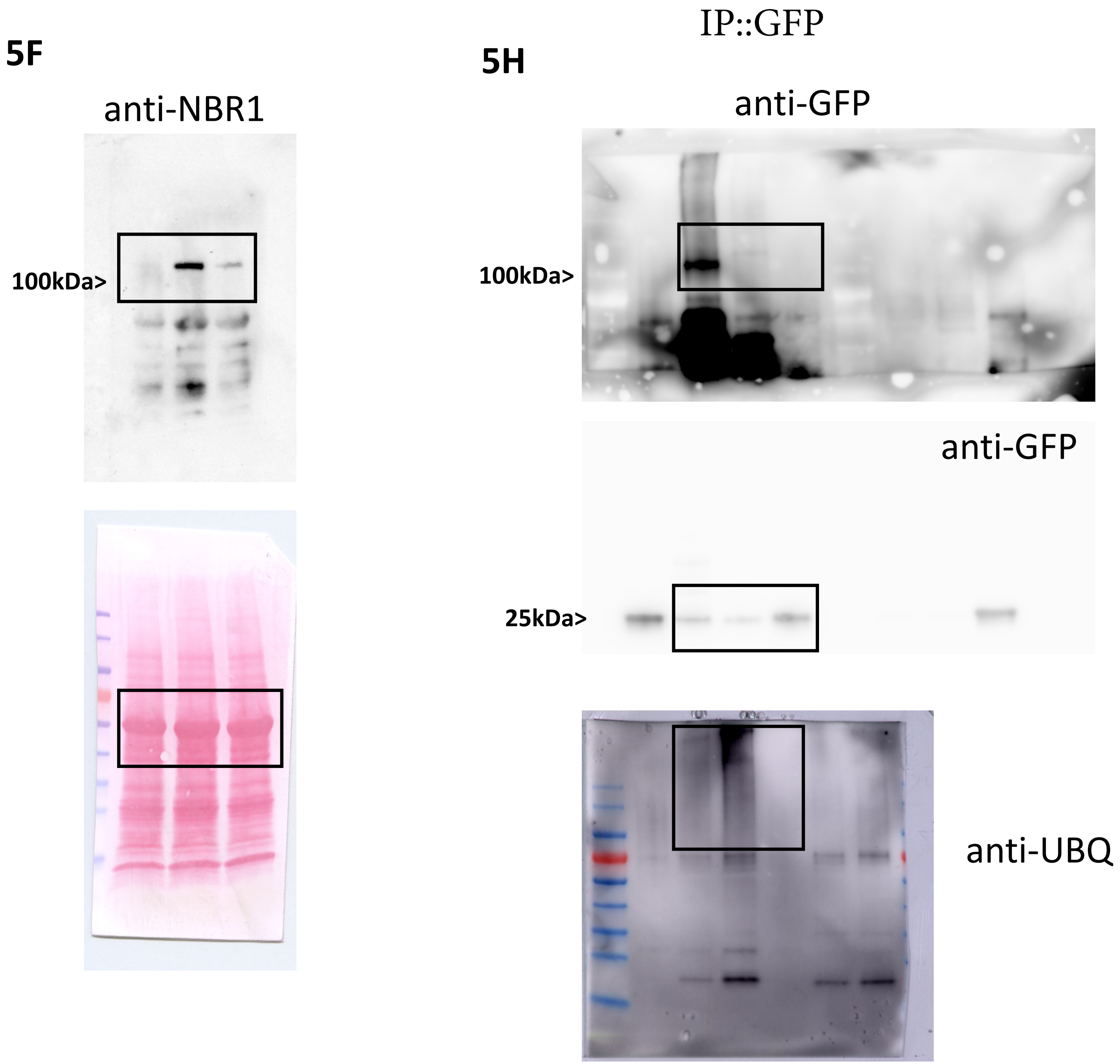
5F
anti-NBR1
100kDa>
IP::GFP
5H
anti-GFP
100kDa>
anti-GFP
25kDa>
anti-UBQ
5J
100kDa>
anti-GFP

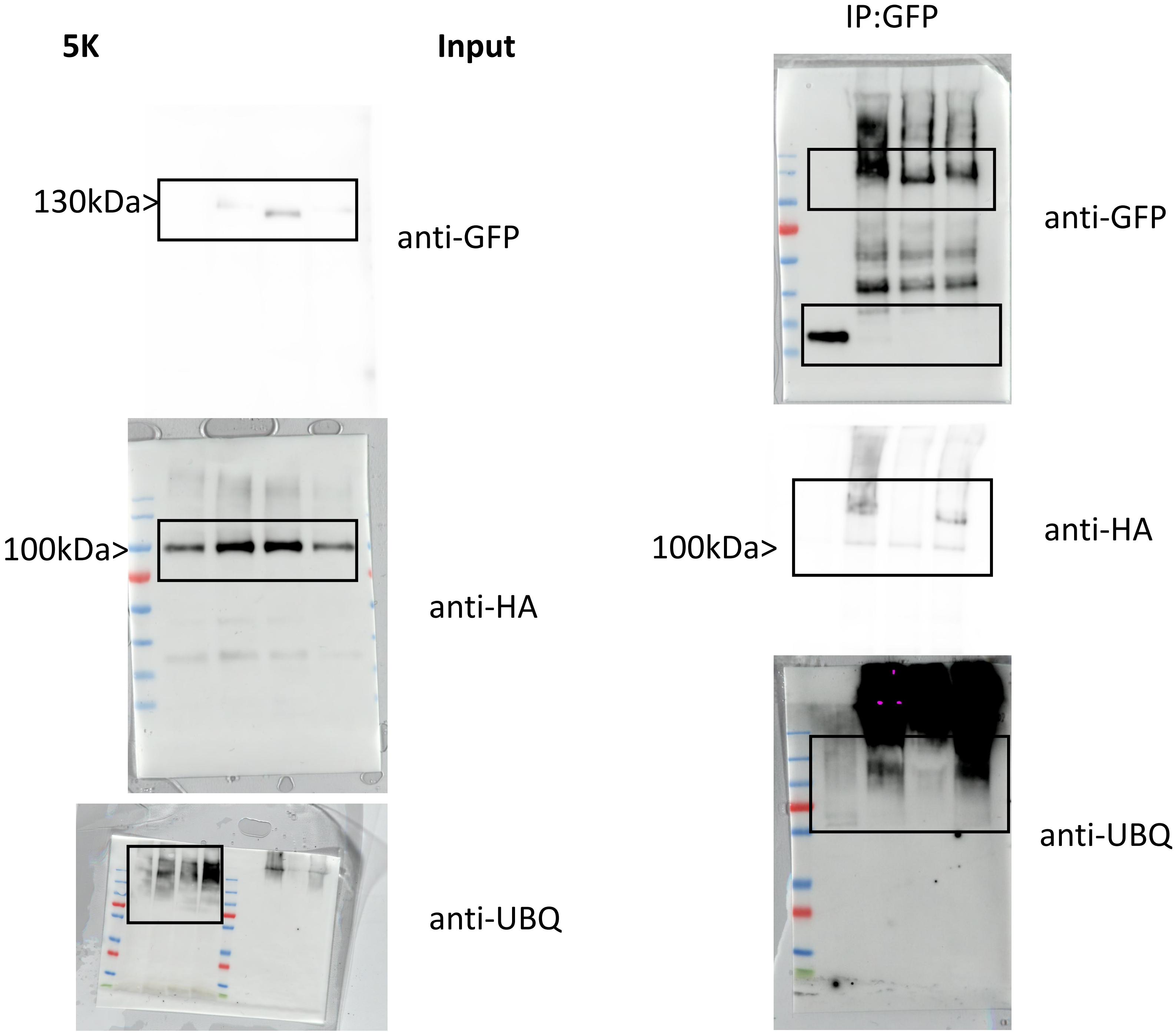

5K
Input
130kDa>
anti-GFP
100kDa>
anti-HA
anti-UBQ
IP:GFP
anti-GFP
100kDa>
anti-HA
anti-UBQ
