# Supplementary material for: A bacterial effector counteracts host autophagy by promoting degradation of an autophagy component
Source: EMBO J. 2022 May 27;41(13):e110352. doi: 10.15252/embj.2021110352 (PMC9251887; doi:10.15252/embj.2021110352)
Supplement: Supplementary file 8 [file EMBJ-41-e110352-s001.pdf]

THE EMBO JOURNAL

A bacterial effector counteracts host autophagy by promoting degradation of an autophagy component

Jia Leong, Margot Raffeiner, Daniela Spinti, Gautier Langin, Mirita Franz-Wachtel, Andrew Guzman, Jung-Gun Kim, Pooja Pandey, Alyona Minina, Boris Macek, Anders Hafrén, Tolga Bozkurt, Mary Beth Mudgett, Frederik Boernke, Daniel Hofius, and Suayib Ustun
DOI: 10.15252/embj.2021110352

Corresponding author(s): Suayib Ustun (suayib.uestuen@zmbp.uni-tuebingen.de)

Review Timeline:

Submission Date:	15th Dec 21
Editorial Decision:	11th Jan 22
Revision Received:	7th Mar 22
Editorial Decision:	30th Mar 22
Revision Received:	20th Apr 22
Accepted:	21st Apr 22

Editor: William Teale

Transaction Report:

(Note: With the exception of the correction of typographical or spelling errors that could be a source of ambiguity, letters and reports are not edited. Depending on transfer agreements, referee reports obtained elsewhere may or may not be included in this compilation. Referee reports are anonymous unless the Referee chooses to sign their reports.)

Dear Dr. Uestuen,

Thank you again for the submission of your manuscript entitled "A bacterial effector counteracts host autophagy by promoting degradation of an autophagy component". We have now received the reports from the referees, which I have copied to the bottom of this message.

As you can see from their comments, all referees appreciate the significance of your work and are generally supportive of publication. Therefore, based on this overall interest, I would like to invite you to address the comments of all referees in a revised version of the manuscript. However, as you will see, each referee raises a substantial list of major issues which will undoubtedly require further experimental input before we can progress. I think it would be a good idea to arrange a Zoom call together at your convenience to allow us to discuss your revision plan and make sure no misunderstandings happen. Please indicate when you might be able to make yourself available.

I should add that it is The EMBO Journal policy to allow only a single major round of revision and that it is therefore important to resolve these concerns at this stage.

I would also like to point out that as a matter of policy, competing manuscripts published during this period will not be taken into consideration in our assessment of the novelty presented by your study ("scooping" protection). We have extended this 'scooping protection policy' beyond the usual 3 month revision timeline to cover the period required for a full revision to address the essential experimental issues. Please contact me if you see a paper with related content published elsewhere to discuss the appropriate course of action.

When preparing your letter of response to the referees' comments, please bear in mind that this will form part of the Review Process File, and will therefore be available online to the community. For more details on our Transparent Editorial Process, please visit our website: https://www.embopress.org/page/journal/14602075/authorguide#transparentprocess

Again, please contact me at any time during revision if you need any help or have further questions.

Thank you very much again for the opportunity to consider your work for publication. I look forward to your revision.

Best regards,

William Teale

William Teale, Ph.D.
Editor
The EMBO Journal

When submitting your revised manuscript, please carefully review the instructions below and include the following items:

1) a .docx formatted version of the manuscript text (including legends for main figures, EV figures and tables). Please make sure that the changes are highlighted to be clearly visible.

2) individual production quality figure files as .eps, .tif, .jpg (one file per figure).

3) a .docx formatted letter INCLUDING the reviewers' reports and your detailed point-by-point response to their comments. As part of the EMBO Press transparent editorial process, the point-by-point response is part of the Review Process File (RPF), which will be published alongside your paper.

4) a complete author checklist, which you can download from our author guidelines (https://wol-prod-cdn.literatumonline.com/pb-assets/embo-site/Author Checklist%20-%20EMBO%20J-1561436015657.xlsx). Please insert information in the checklist that is also reflected in the manuscript. The completed author checklist will also be part of the RPF.

5) Please note that all corresponding authors are required to supply an ORCID ID for their name upon submission of a revised manuscript.

6) We require a 'Data Availability' section after the Materials and Methods. Before submitting your revision, primary datasets produced in this study need to be deposited in an appropriate public database, and the accession numbers and database listed under 'Data Availability'. Please remember to provide a reviewer password if the datasets are not yet public (see https://www.embopress.org/page/journal/14602075/authorguide#datadeposition). If no data deposition in external databases is

needed for this paper, please then state in this section: This study includes no data deposited in external repositories. Note that the Data Availability Section is restricted to new primary data that are part of this study.

Note - All links should resolve to a page where the data can be accessed.

7) When assembling figures, please refer to our figure preparation guideline in order to ensure proper formatting and readability in print as well as on screen:
http://bit.ly/EMBOPressFigurePreparationGuideline

Please remember: Digital image enhancement is acceptable practice, as long as it accurately represents the original data and conforms to community standards. If a figure has been subjected to significant electronic manipulation, this must be noted in the figure legend or in the 'Materials and Methods' section. The editors reserve the right to request original versions of figures and the original images that were used to assemble the figure.

8) For data quantification: please specify the name of the statistical test used to generate error bars and P values, the number (n) of independent experiments (specify technical or biological replicates) underlying each data point and the test used to calculate p-values in each figure legend. The figure legends should contain a basic description of n, P and the test applied. Graphs must include a description of the bars and the error bars (s.d., s.e.m.).

9) We would also encourage you to include the source data for figure panels that show essential data. Numerical data can be provided as individual .xls or .csv files (including a tab describing the data). For 'blots' or microscopy, uncropped images should be submitted (using a zip archive or a single pdf per main figure if multiple images need to be supplied for one panel). Additional information on source data and instruction on how to label the files are available at .

10) We replaced Supplementary Information with Expanded View (EV) Figures and Tables that are collapsible/expandable online (see examples in https://www.embopress.org/doi/10.15252/embj.201695874). A maximum of 5 EV Figures can be typeset. EV Figures should be cited as 'Figure EV1, Figure EV2" etc. in the text and their respective legends should be included in the main text after the legends of regular figures.

- For the figures that you do NOT wish to display as Expanded View figures, they should be bundled together with their legends in a single PDF file called *Appendix*, which should start with a short Table of Content. Appendix figures should be referred to in the main text as: "Appendix Figure S1, Appendix Figure S2" etc. See detailed instructions regarding expanded view here: .

- Additional Tables/Datasets should be labelled and referred to as Table EV1, Dataset EV1, etc. Legends have to be provided in a separate tab in case of .xls files. Alternatively, the legend can be supplied as a separate text file (README) and zipped together with the Table/Dataset file.

11) Our journal encourages inclusion of *data citations in the reference list* to directly cite datasets that were re-used and obtained from public databases. Data citations in the article text are distinct from normal bibliographical citations and should directly link to the database records from which the data can be accessed. In the main text, data citations are formatted as follows: "Data ref: Smith et al, 2001" or "Data ref: NCBI Sequence Read Archive PRJNA342805, 2017". In the Reference list, data citations must be labeled with "[DATASET]". A data reference must provide the database name, accession number/identifiers and a resolvable link to the landing page from which the data can be accessed at the end of the reference. Further instructions are available at .

Referee #1:

In this manuscript, Leong et al. studied the role of autophagy during Xcv infection. They have identified a type 3 effector protein XopL as a major regulator of autophagic responses. They went further and showed that XopL interacts with SH3P2, a highly conserved protein that has been suggested to play a role in autophagy and vacuolar trafficking. Further characterization of XopL has shown that the effector itself is targeted by autophagy via the NBR1 pathway, suggesting a complex interplay between host defense responses and effector function. Overall, it is an exciting story that highlights the elaborate arms race underlying autophagy-mediated antibacterial defense responses. I think it will be exciting for the broad readership of the EMBO journal as similar mechanisms may be involved in other host-microbe interaction systems.

Below is a list of suggestions and questions, which I hope will help improve the manuscript.

Writing and data presentation related (minor suggestions):

1. I think "unprecedented" is oversold in this scenario since the author's previous studies have shown similar mechanisms are in play in viruses. They should modify it to restrict it to bacterial pathogens maybe. "Our results implicate plant antimicrobial autophagy in depletion of a bacterial virulence factor and unravels an unprecedented pathogen strategy to counteract defense-related autophagy."

2. Line 104-110- this paragraph is crucial for the readers, but the current form is quite difficult to follow. Can the authors explain their findings step by step in a simple manner and help the readers understand the logic of their discovery?

3. Line122-165- I do appreciate that the authors have used various assays to measure autophagic degradation, but this paragraph is way too long and very difficult to follow in its current form. The readers would really benefit from prompters like, "We used x number of assays to measure autophagy. 1st..., 2nd ....

4. Fig1A- could it help to have insets of the images where we can see the effect of Xcv infection on autophagosome numbers clearly?

5. The microscopy images showing induction of autophagosomes were taken at 2dpi, whereas the atg7 silencing was taken at 6 hpi. Why are there time differences? It would be great to somehow explain these at least in the materials and methods sections.

6. Fig2B-seems the authors split the GFP and GFP-XopL blots. I assume these are from the same blots, so please don't split them, lets us see the whole blot. The same applies to many other blots. Please don't split these blots.

7. Fig2C-why use GUS as an empty vector control? Should not the authors use a GFP empty vector?

8. Fig2 title: XopL suppresses autophagic degradation, not autophagy. they clearly see increased autophagosome formation, but reduced vacuolar degradation. So, please adjust the title accordingly, as you have been rightly describing throughout the text.

9. Line 296-Re- no known targets of XopL- Ma et al (https://doi.org/10.1186/s42483-020-00055-w) seem to suggest at least one more target? Also, Erickson et al., 2017 (https://doi.org/10.1111/tpj.13813) suggest a microtubule localization. The authors should adjust this statement and discuss these papers in the discussion part.

10. Fig3B-please do not split the blots or at least make it very clear for the readers that these are the same blots just split for visualization purposes.

11. Line 106-why is Xcv underlined?

12. Line 133-AZD treatment, please include a reference to show it induces autophagy

13. Line 357-Fig3 title: XopL interacts with and degrades SH3P2 and to boost Xcv virulence should be XopL interacts with and degrades SH3P2 to boost Xcv virulence

14. Line 426-Fig 4 title: "XopL mediates the proteasome degradation of SH3P2 via its E3 ligase activity" should be "XopL mediates the proteasomal degradation of SH3P2 via its E3 ligase activity"

15. Fig5H-Fig5K: maybe the authors could split these findings to another figure? There is no NBR1 in these results and they are a bit different than the other findings of this figure.

Experimental (Requires additional experiments):

16. Fig3E-Re-reduced protein levels: does XopL cause cell death? Can the authors measure levels of some other proteins, as ponceau staining is not that sensitive, to make sure that they are not looking at the effect of cell death?

17. FigS8D- why do we see input MBP and pull down MBP in different sizes?

18. Fig4- the authors define a very nice control for the effector XopL that lacks E3 ligase activity. However, I can't find any assays where they assess the stability of this mutant? In C and F, it seems less stable? If it has stability issues, how can they use it as a control? Could they please clarify this?

19. FigS11B- This is a crucial experiment but I think the assay design is problematic. In the lane where the authors have the XopL mutant, there is no ATP, so ubiquitination can't happen anyways. They need to compare XopL and the mutant in the presence of ATP. Also, why do they have a lane without any GST expressing protein (second to last)?

20. Fig5D- in the previous figures the authors showed that XopL increases the protein levels of NBR1, due to a blockage in autophagic degradation. In this experiment, we don't see that difference? Also in Fig5E, we seem to have more NBR1 in XopL mutant, which would be in favor of wild type inducing cell death?

21. In Fig5H- in the GFP-IP, why are we not seeing the XopL band? Also in the input, the levels seem to be quite different. Could the authors not normalize these protein levels? Otherwise, it is very difficult to assess these results.

22. Is the self-ubiquitination dependent on the E3 ligase activity? Why not use the XopL mutant that lacks the ligase activity as a control instead of GFP alone? I find the K191 related results quite confusing. The XopL E3 ligase mutant still undergoes autophagy as shown in Fig S17, but K191 is reduced in ubiquitination. I don't see how these findings fit together.

Referee #2:

In the manuscript "A bacterial effector counteracts host autophagy by promoting degradation of an autophagy component" by Leong and colleagues, authors show that the bacterial effector XopL interacts with two host proteins, namely NBR1 and SH3P2 to modulate autophagy.
Through a targeted screen authors show the potential involvement of XopL in the inhibition of host autophagy, which they subsequently confirm. They go on to identify SH3P2, which has been shown to participate in autophagy, as a target of XopL. XopL mediates the degradation of SH3P2 via the proteasome. Finally, they show that XopL also interacts with the autophagy receptor NBR1, and propose that it mediates vacuolar degradation of XopL via autophagy.
There has been a great deal of controversy surrounding the role of autophagy during the immune response in plants. As such the manuscript by Leong and colleagues not only sheds light on the function of different host components involved in autophagy during the plant immune response, but it also reveals a pathogen effector that interferes with these cellular processes. As such this is a timely and relevant study which will draw the attention of a wide audience.
However, some of the authors conclusions, in particular the proposed role of NBR1 in the clearance of XopL, need further experimental validation.
Major issues
1. Although the link between SH3P2 and XopL is well characterized, there are some issues that require clarification or improvement:
-To confirm the in vivo interaction between AtSH3P2 and XopL, authors overexpressed both proteins in N. benthamiana. In the IP blot however, there is no band corresponding to the size of AtSH3P2-HA (Figure 3B). Also, what does the asterisk stand for?
-The evidence that XopL ubiquitinates SH3P2 needs to be improved. Even though there are some extra bands visible, these do not correspond to a pattern of polyubiquitination of SH3P2. Moreover, authors include an inactive XopL variant, but in a separate blot and with (what seems like) significantly different levels of SH3P2. Hence, these two results are not comparable. Authors need to show a direct comparison.
-It would also help their case to demonstrate that XopL activity results in ubiquitinated SH3P2 in vivo.
2. Coexpression of XopL with NBR1 results in reduced levels of NBR1, and together with the shown interaction between both, leads authors to propose that NBR1 targets XopL. In their abstract authors state to unravel an unprecedented pathogen strategy. However, the presented data does not fully support this hypothesis.
From an evolutionary perspective it's extremely unlikely that an evolutionary highly conserved protein as NBR1, present in most (if not all) plants, would have evolved to target one effector from a specialized pathogen type. The evolutionary dynamics of effectors should exceed by far that of a gene with core functions. In fact, Figure 5E seems to suggest that coexpression of XopL stabilizes NBR1 in an E3 ligase activity independent manner. Can authors exclude that XopL affects NBR1 function? Can it be excluded that XopL is a suicidal effector? The fact that XopL degradation may be mediated by NBR1, does not imply a directionality of the interaction. As mentioned above, XopL may interact with NBR1 and impair its function, while at the same time being itself degraded. Moreover, authors indeed show that XopL impairs autophagic flux, which should also inhibit its own degradation.
Along the same lines, although authors showed a physical interaction between NBR1 and XopL, they investigate the possibility that NBR1 also associates to XopL by means of ubiquitin chains attached to itself. Even it makes perfect sense, it is counter-intuitive to the previous observation of physical interaction. Author could try to better introduce the rational or the mechanism they hypothesize. For instance, that NBR1 is first recruited by ubiquitin chains that offer larger interaction surface (because repetitive) that leads to a subsequent direct interaction.
-Following the hypothesis that NBR1 mediates the degradation of XopL, authors test whether XopL is able to autoubiquitinate, which would potentially contribute to both its degradation, as well as the recruitment of NBR1. A previous study however, failed to show autoubiquitination of a truncated variant of XopL (singer et al. 2013). The in vivo data seems to support autoubiquitination (while adding the molecular size markers would help data interpretation, please add throughout). However, the in vitro assay is again not convincing. This may however be due to the figure which is of low quality and small (Figure S15B). Please provide a high-resolution image.
3. Authors use a Luc-ATG8/NBR1 fusion to test autophagic flux and show that inoculation with Xcv, or the overexpression of XopL, result in an accumulation of both reporters. To support their claims, authors should include a positive control to show that

accumulatio of the reporter is not due to overloading of the system under the used conditions, particularly by overexpression. This could be done simply by including HopM1 and/or AZD treatment.
Related to this, in Figure S4B treatment with MG132 inhibited autophagy, which is unexpected. Would the proteotoxic effect not rather activate autophagy? Please comment.
4. Authors state that ATG8 levels are not further increased after ConA treatment, but surprisingly, there is rather a decrease in ATG8-labelled bodies (Figure 2C) and ATG8 (Figure 2D) protein after ConA treatment. This requires clarification.
5. Related to this point, ConA treatment would be expected to result in the accumulation of autophagic bodies in the vacuole. This is however not the case according to Figure 1A, or other data presented in this manuscript. Is this a N. benthamiana-specific phenomena? Please comment.
6. The legend to Figure 4F is missing. Authors should also repeat this experiment, or reload the sample since there were obvious problems during the electrophoresis, which go beyond simple aesthetic problems and do not allow to confirm equal loading.
7. A more general point: The Rubisco band, even used commonly to asses equal loading, is utterly unappropriated for this purpose due to its inherent "overloading", because it's so abundant. Please include additional portions to include bands above and below, with less abundant bands to properly assess loading.
Minor points
- I would suggest to simply use NBR1 and not NBR1/Joka or Joka, since NBR1 is the common and more recognized name of the protein.
-Lane #49 please include reference
-lane #469 ConA blocks vacuolar degradation, not autophagy. Please edit.
- Lane#479 Authors performed an IP not a pull-down.
- lane 697 There have been reports of effectors with autoubiquitination activity e.g. AvrPtoB form Pseudomonas

Referee #3:

The manuscript entitled "A bacterial effector counteracts host autophagy by promoting degradation of an autophagy component" reported a mutual regulatory role of autophagy in Xanthomonas-plant interaction. The authors show that a T3E XopL from Xanthomonas can suppress plant autophagy, likely by mediating the ubiquitination and degradation of a plant autophagy component SH3P2, and on the other hand, the protein level of XopL is also down-regulated by NBR1/Joka2-mediated selective autophagy. While the topic is interesting and the authors presented a large amount of data to demonstrate the molecular interplays between Xanthomonas effector and plant autophagy machinery, the interpretations of some key results are confusing and warrant clarification. Also, additional experiments are required to strengthen the major conclusions of the paper.

Specific comments:
1. Formation of autophagic structures and upregulation of ATG/NBR1 genes are usually associated with activation of autophagy, as published by previous studies including one from the corresponding author (Üstün et al., Plant Cell 2018). However, the authors claim that Xanthomonas (and later on XopL) blocks autophagy, although an increase of ATG8g-labelled autophagosomes and ATG/NBR1 expression was observed (Figure 1 and 2). This is somewhat counterintuitive. An alternative interpretation could be that Xanthomonas infection induces autophagy and XopL is one of the major inducers. There are other assays, such as detection of processed ATG8 protein or release of free GFP from GFP-ATG8 protein, that people routinely perform to indicate the activity of autophagy in planta and should be tested here during Xanthomonas infection. In Figure 1A, the increase of autophagic structures by ConA treatment (in the mock panels) seems fairly minor. Also, the inoculation approach (dipping or infiltration, at what concentration) is not clearly described in the legend. 2dpi is a relatively late point. How early can the autophagic structures be observed?

2. What is a general role of autophagy to Xcv infection? In Fig. 1E and S2A, suppression of autophagy only caused subtle difference in bacterial multiplication. This questions the importance of autophagic activity in plant-Xcv interaction. In addition, what is the phenotype of Xcv ΔxopQ ΔxopL infection under these treatments?

3. The authors used different autophagy inhibitors in different assays, which is confusing. What is the difference between ConA and AIMp, in terms of inhibiting autophagy and the underlying mechanisms? A more detailed description is needed to explain why one, but not the other, is used in particular assays. This helps readers that do not directly work in the autophagy field.

4. Figure 3: 3B, which band indicates SH3P2-HA in the IP samples? In figure 3B-E, using SH3P2 from Arabidopsis in the interaction and protein degradation assays is odd, since all other bacteria-related assays were performed in tomato or tobacco plants. Do all SH3P2 homologs from tomato or tobacco have expression problems? What about in vitro? It is important to show the XopL interaction with and protein degradation effect on tomato or tobacco SH3P2.

5. Transient over-expression of bacterial effectors is prone to artifact, does XopL reduce SH3P2 protein during bacterial inoculation (e.g., comparing Xcv and XcvΔxopL mutant strain)? Is the ubiquitination level of SH3P2 protein altered during bacterial infection, comparing Xcv and XcvΔxopL mutant strain?

6. XopL has E3 ligase activity. Does it induce ubiquitination and degradation of NBR1, since they also interact and XopL, SH3P2 and NBR1 could be in the same protein complex?

7. Figure 5: Again, transient expression of effectors in tobacco could cause artificial results. It should be shown whether XopL protein amount is changed in NBR1-silenced/mutated plants during Xanthomonas infection.

8. NBR1/Jokes2 interacts with both GFP-XopL and GFP-XopLΔE3 (Figure 5E). Since XopLΔE3 is a point mutant of 3 amino acids, do these mutations affect its interaction with substrates? If not, NBR1/Jokes2 may also mediate the degradation of other XopL target proteins in the complex. The statements in lines 484-486 may be inappropriate.

Minor concerns:
1. In line 123, there is a citation with inconsistent format.
2. Line 456, Fig S5A did not show XopL protein accumulation under ConA treatment.
3. Lines 248: the "normalized RLUC-NBR1/FLUC" under Xcv ΔxopQ ΔxopL treatment seems dramatically higher than mock treatment. Therefore , "Xcv ΔxopQ ΔxopL still leads to a slight increase in both RLUC-ATG8a/FLUC and RLUC-NBR1/FLUC ratios..." may not be appropriate.
4. Figure S7C: the leaf shape does not look like epidermal cells. What does the purple color indicate?
5. Figure 4F: the bands look ugly, especially in PS-stained blot. There is no legend for it.

Point-by-point response to editor and reviewers' comments on the manuscript by Leong et al.

Referee #1:

In this manuscript, Leong et al. studied the role of autophagy during Xcv infection. They have identified a type 3 effector protein XopL as a major regulator of autophagic responses. They went further and showed that XopL interacts with SH3P2, a highly conserved protein that has been suggested to play a role in autophagy and vacuolar trafficking. Further characterization of XopL has shown that the effector itself is targeted by autophagy via the NBR1 pathway, suggesting a complex interplay between host defense responses and effector function. Overall, it is an exciting story that highlights the elaborate arms race underlying autophagy-mediated antibacterial defense responses. I think it will be exciting for the broad readership of the EMBO journal as similar mechanisms may be involved in other host-microbe interaction systems.

Below is a list of suggestions and questions, which I hope will help improve the manuscript.

Writing and data presentation related (minor suggestions):

1. I think "unprecedented" is oversold in this scenario since the author's previous studies have shown similar mechanisms are in play in viruses. They should modify it to restrict it to bacterial pathogens maybe. "Our results implicate plant antimicrobial autophagy in depletion of a bacterial virulence factor and unravels an unprecedented pathogen strategy to counteract defense-related autophagy."

We agree with this comment and added "in plant-bacteria interactions".

2. Line 104-110- this paragraph is crucial for the readers, but the current form is quite difficult to follow. Can the authors explain their findings step by step in a simple manner and help the readers understand the logic of their discovery?

We have changed this in the revised version.

3. Line122-165- I do appreciate that the authors have used various assays to measure autophagic degradation, but this paragraph is way too long and very difficult to follow in its current form. The readers would really benefit from prompters like, "We used x number of assays to measure autophagy. 1st..., 2nd ....

Thank you for this suggestion. The paragraph seems rather long as we try to explain the choice of experiments and follows the structure of the figure.

4. Fig1A- could it help to have insets of the images where we can see the effect of Xcv infection on autophagosome numbers clearly?

The images are only there to visualize that we did not take a "small" given area to quantify our images. Insets are not really "adding" anything to this figure.

5. The microscopy images showing induction of autophagosomes were taken at 2dpi, whereas the atg7 silencing was taken at 6 hpi. Why are there time differences? It would be great to somehow explain these at least in the materials and methods sections.

We show in the same supplemental figure S1B as well as Fig2D that Xcv induces a block of autophagic degradation at earlier time-points. The atg7 silencing experiment was done to confirm that we are looking into autophagic structures (this was suggested by a previous reviewer). As reviewer 3 pointed out, 2dpi can be considered as a later time point during infection (although we do not see any tissue collapse) and for this reason we included earlier time points.

6. Fig2B-seems the authors split the GFP and GFP-XopL blots. I assume these are from the same blots, so please don't split them, lets us see the whole blot. The same applies to many other blots. Please don't split these blots.

We have done this out of "aesthetic" reasons, as we have many sub figures in our Figure panels. The "free GFP" signal is always much stronger than the full-length signal of XopL. We have to use longer exposures to detect XopL and thus the "freeGFP" part is oversaturated and not suitable as a figure. If requested, we can provide the original blots in the supplemental. As requested by the referee we will indicate this in the figure.

7. Fig2C-why use GUS as an empty vector control? Should not the authors use a GFP empty vector?

We have shown in multiple assays (e.g. Fig 2B) that GFP does not increase ATG8 abundance. As GUS-HA is at a similar size than XopL it can be also considered as a valid control.

8. Fig2 title: XopL suppresses autophagic degradation, not autophagy. they clearly see increased autophagosome formation, but reduced vacuolar degradation. So, please adjust the title accordingly, as you have been rightly describing throughout the text.

Autophagy refers to autophagy as a whole process including importantly its end point (degradation), so a block of autophagy is accurate here. At this stage we do not really know if the formed autophagosomes are really "complete"/mature. As proposed before, SH3P2 might have a role in the late steps of autophagosome formation or in the fusion (Zhuang et al., 2013).

9. Line 296-Re- no known targets of XopL- Ma et al (https://doi.org/10.1186/s42483-020-00055-w) seem to suggest at least one more target? Also, Erickson et al., 2017 (https://doi.org/10.1111/tpj.13813) suggest a microtubule localization. The authors should adjust this statement and discuss these papers in the discussion part.

Ma et al., are working on XopL from Xoo-Rice and we are working XopL from Xcv-Tomato/Pepper. We think that the targets might be different because of different host systems. We have discussed the possible microtubule localization of the E3 ligase dead version of XopL in the ms (l694-699), p. 17.

10. Fig3B-please do not split the blots or at least make it very clear for the readers that these are the same blots just split for visualization purposes.

We have now indicated this for the readers.

11. Line 106-why is Xcv underlined?

Corrected.

12. Line 133-AZD treatment, please include a reference to show it induces autophagy

We have included a reference.

13. Line 357-Fig3 title: XopL interacts with and degrades SH3P2 and to boost Xcv virulence should be XopL interacts with and degrades SH3P2 to boost Xcv virulence

We have changed this accordingly.

14. Line 426-Fig 4 title: "XopL mediates the proteasome degradation of SH3P2 via its E3 ligase activity" should be "XopL mediates the proteasomal degradation of SH3P2 via its E3 ligase activity"

We have changed this accordingly.

15. Fig5H-Fig5K: maybe the authors could split these findings to another figure? There is no NBR1 in these results and they are a bit different than the other findings of this figure.

We show that NBR1 is still interacting with the K191A mutant of XopL. The reason why the ubiquitination blots are in this figure is that NBR1 recognizes and degrades ubiquitinated proteins. However, we have performed a new co-IP showing that XopL might interact with Joka2 in a ubiquitin-dependent and independent manner (Figure 5K). The other figure was moved to the supplemental (S16E).

Experimental (Requires additional experiments):

16. Fig3E-Re-reduced protein levels: does XopL cause cell death? Can the authors measure levels of some other proteins, as ponceau staining is not that sensitive, to make sure that they are not looking at the effect of cell death?

In our hands XopL never induces cell death. Singer et al., 2013 indeed report that XopL has cell death activity, which we never observed (see figure below). One difference is that they see cell death at later time-points (4dpi and 6dpi) and we use 1 and 2 dpi for all our assays. Also, from their quantification of ion leakage at 2dpi (Fig 1B) you can see that there is no tissue damage induced by XopL.

Figure for Referee not shown.

We have multiple blots in the manuscript showing that protein amounts do not always decrease in the presence of XopL (e.g., ATG8, NBR1 blots) In Fig S17A you can see those levels of GFP are unchanged and that RFP-AIMp is rather "increased" in the presence of XopL. FigS10 also shows that SH3P2 transcripts are rather increased in the presence of XopL, arguing against a "cell death" effect of XopL. Below you will find another blot showing that XopL does not decrease the abundance of the RFP-AIMp fusion protein. We have no included a blot showing that XopL degrades SH3P2 while it does not change the protein levels of Histone H3 (Supp Figure S8E).

Figure for Referee not shown.

17. FigS8D- why do we see input MBP and pull down MBP in different sizes?

The input sample is the GST fusion protein (MBP protein is bound to amylose matrix before we incubate it with the "input"). The band in the MBP blot is a cross-reaction. We will mark this in the revised version.

18. Fig4- the authors define a very nice control for the effector XopL that lacks E3 ligase activity. However, I can't find any assays where they assess the stability of this mutant? In C and F, it seems less stable? If it has stability issues, how can they use it as a control? Could they please clarify this?

With this experiment we confirm our in vitro data that SH3P2 is degraded by XopL dependent on its E3 ligase activity. We are aware that the E3 ligase mutant is sometimes less stable than XopL but this might be due to its lost ability to block autophagy. Thus, it is more prone to get degraded by autophagy. Therefore, we provide in vitro ubiquitination assays with deltaE3, in which we show it lacks the ability to generate polyubiquitin chains and hence is not trans-ubiquitinating SH3P2 (Fig S11B).

19. FigS11B- This is a crucial experiment but I think the assay design is problematic. In the lane where the authors have the XopL mutant, there is no ATP, so ubiquitination can't happen anyways. They need to compare XopL and the mutant in the presence of ATP. Also, why do they have a lane without any GST expressing protein (second to last)?

The first lane has all components (including ATP) needed for the in vitro ubiquitination assay. "No ATP" lane is a control as the reaction can only occur in presence of ATP. We also have a control without any XopL (GST fusion) as an additional control that ubiquitination is not mediated by E2 ligases (which in rare cases also happens).

20. Fig5D- in the previous figures the authors showed that XopL increases the protein levels of NBR1, due to a blockage in autophagic degradation. In this experiment, we don't see that difference? Also in Fig5E, we seem to have more NBR1 in XopL mutant, which would be in favor of wild type inducing cell death?

It is true that in input lanes, Joka2 is not increased in GFP-XopL compared to GFP (Fig. 5D). However, most of our experiments involving GFP-XopL and NBR1/Joka2 (2B, 5E, 5K and repetitions) shows the accumulation of NBR1/Joka2 during XopL expression. The reason for this difference might be the buffer composition and several centrifugation steps during the IP experiment. As the aim of the experiment in Fig. 5D is to show that GFP-XopL

associates with NBR1/Joka2 we will keep the figure as it is. We also want to mention again that XopL is not inducing cell death in our hands (see response above).

21. In Fig5H- in the GFP-IP, why are we not seeing the XopL band? Also in the input, the levels seem to be quite different. Could the authors not normalize these protein levels? Otherwise, it is very difficult to assess these results.

The XopL band is present in the IP sample. We have marked this with an asterisk in the revised version. We did not want to expose the blot longer, because of the sample on the left side. It is hard to normalize protein levels as the effect of autophagy inhibitor AIMp on the deltaE3 version is sometimes stronger than on the XopL WT version due to a possible higher autophagic flux of XopL deltaE3. Thus, the difference in protein abundance of XopL and its mutant when coexpressed with RFP-AIMp is important information, as it shows that XopLΔE3 might be more subject to autophagic degradation than its WT counterpart. We think that normalization is not needed as probing the IP:GFP samples with anti-UBQ clearly shows increased ubiquitination of XopL WT even if the its protein amount is lower. In other words, increasing XopL WT protein would increase even more the ubiquitination shown, which does not undermine our assessment of these results.

22. Is the self-ubiquitination dependent on the E3 ligase activity? Why not use the XopL mutant that lacks the ligase activity as a control instead of GFP alone? I find the K191 related results quite confusing. The XopL E3 ligase mutant still undergoes autophagy as shown in Fig S17, but K191 is reduced in ubiquitination. I don't see how these findings fit together.

We have identified the K191 ubiquitination site in vivo and in vitro, which means that the self-ubiquitination site is indeed dependent on its E3 ligase activity. However, based on Fig5H and K we think that there might be also other ubiquitination sites present in XopL that are targeted by host E3 ligases. It is also possible that mutating K191 leads to the ubiquitination of other sites which would usually not be ubiquitinated. We have indeed indications that several other ubiquitination sites are present in XopL and that XopL might associate with plant E3 ligases. We are understandably interested in elucidating the underlying mechanism, but this is a substantial independent line of research that we feel is beyond the scope of the current manuscript.

Referee #2:

In the manuscript "A bacterial effector counteracts host autophagy by promoting degradation of an autophagy component" by Leong and colleagues, authors show that the bacterial effector XopL interacts with two host proteins, namely NBR1 and SH3P2 to modulate autophagy.
Through a targeted screen authors show the potential involvement of XopL in the inhibition of host autophagy, which they subsequently confirm. They go on to identify SH3P2, which has been shown to participate in autophagy, as a target of XopL. XopL mediates the degradation of SH3P2 via the proteasome. Finally, they show that XopL also interacts with the autophagy receptor NBR1, and propose that it mediates vacuolar degradation of XopL via autophagy.
There has been a great deal of controversy surrounding the role of autophagy during the immune response in plants. As such the manuscript by Leong and colleagues not only sheds light on the function of different host components involved in autophagy during the plant immune response, but it also reveals a pathogen effector that interferes with these cellular processes. As such this is a timely and relevant study which will draw the attention of a wide audience.
However, some of the authors conclusions, in particular the proposed role of NBR1 in the clearance of XopL, need further experimental validation.
Major issues
1. Although the link between SH3P2 and XopL is well characterized, there are some issues that require clarification or improvement:
-To confirm the in vivo interaction between AtSH3P2 and XopL, authors overexpressed both proteins in N. benthamiana. In the IP blot however, there is no band corresponding to the size of AtSH3P2-HA (Figure 3B). Also, what does the asterisk stand for?
Our response:
After re-examining our blots, we realized that the asterisk, which indicates the SH3P2 full length protein was placed wrong. We have corrected this. We hypothesize that the lower molecular weight bands of SH3P2-HA might be modified forms (e.g., cleavage/degradation products).
-The evidence that XopL ubiquitinates SH3P2 needs to be improved. Even though there are some extra bands visible, these do not correspond to a pattern of polyubiquitination of SH3P2.
Our response:
We are very sure that the extra bands correspond to a pattern of polyubiquitinated SH3P2 as the majority (we have marked it now) is missing in the control samples. The three major other bands are also present in the control lanes and thus do not correspond ubiquitinated SH3P2. We also performed IP-MS MS in vitro and in vivo of this sample

and identified that SH3P2 is indeed ubiquitinated at lysine 24 (K24) in the presence of XopL (see figures below). We have also mutated this lysine to alanine to block ubiquitination at this site. Although SH3P2 K24A protein is more stable in planta, it is still degraded by XopL which indicates that XopL ubiquitinates SH3P2 at other sites, and/or that other lysines become available for ubiquitination when lysine 24 is mutated. SH3P2 might also be degraded by plant E3 ligase(s) as mutation of K24 stabilizes effectorSH3P2 even in the absence of XopL. We would like to keep this data for a follow-up publication in the future.

Figure for Referee not shown.

Moreover, authors include an inactive XopL variant, but in a separate blot and with (what seems like) significantly different levels of SH3P2. Hence, these two results are not comparable. Authors need to show a direct comparison.

Our response:

We have used equal amounts of SH3P2 and all other proteins for all in vitro ubiquitination assays (see also material method section). The exposure time of both blots is different (see below, longer exposure of Fig S11B). From the in vitro assay you can also see that the inactive XopL variant does not produce any polyubiquitin chains and thus will be never able to polyubiquitinate SH3P2. It is an additional control that the inactive version of XopL is (i) inactive, (ii) not able to degrade SH3P2 (because it lacks E3 ligase activity), which is not due to reduced stability in planta.

Figure for Referee not shown.

-It would also help their case to demonstrate that XopL activity results in ubiquitinated SH3P2 in vivo.

Our response:

We thank the reviewer for this remark. We have performed this assay previously, but we think it might be tricky to show in vivo ubiquitination: as SH3P2 undergoes proteasomal degradation and ubiquitination even when XopL is

not present. We have now added another supplemental figure (S10A) stating that SH3P2 is a "general" proteasome target. Thus, it will be very difficult to demonstrate XopL-specific ubiquitination in vivo. For this we need to identify the plant E3 ligase that ubiquitinates SH3P2 and perform the assays in the mutant background. We are understandably interested in elucidating the ubiquitination of SH3P2 also by plant E3 ligases, but this is a substantial independent line of research that we feel is beyond the scope of the current manuscript.

Figure for Referee not shown.

2. Coexpression of XopL with NBR1 results in reduced levels of NBR1, and together with the shown interaction between both, leads authors to propose that NBR1 targets XopL. In their abstract authors state to unravel an unprecedented pathogen strategy. However, the presented data does not fully support this hypothesis.
Our response: XopL results in an increase of NBR1 protein levels.
From an evolutionary perspective it's extremely unlikely that an evolutionary highly conserved protein as NBR1, present in most (if not all) plants, would have evolved to target one effector from a specialized pathogen type. The evolutionary dynamics of effectors should exceed by far that of a gene with core functions.
Our response: We don't think that NBR1 is only targeting XopL. It is very likely that NBR1 targets other effectors as it was already shown that NBR1 targets viral proteins (e.g., Hafrén et al., 2018, Plant Physiology). That is why we discuss the concept "effectorphagy" on p18, l681.
In fact, Figure 5E seems to suggest that coexpression of XopL stabilizes NBR1 in an E3 ligase activity independent manner. Can authors exclude that XopL affects NBR1 function? Can it be excluded that XopL is a suicidal effector? The fact that XopL degradation may be mediated by NBR1, does not imply a directionality of the interaction. As mentioned above, XopL may interact with NBR1 and impair its function, while at the same time being itself degraded. Moreover, authors indeed show that XopL impairs autophagic flux, which should also inhibit its own degradation.
Our response: As XopL is blocking autophagic flux it also indirectly interferes with NBR1 function as NBR1 together with its cargos is not degraded anymore (see Fig 2B, S6 etc., also note that NBR1/Joka2 transcripts are not induced at 2 dpi while protein amount is, indicating that NBR1 turnover is impaired by XopL).
We also do discuss this idea in the context of the K191 (self-)ubiquitination site possibly being used by XopL to target itself to the autophagy pathway, in p19 lines 780-87. We are understandably interested in elucidating how XopL affects the function of NBR1, but this is a substantial independent line of research that we feel is beyond the scope of the current manuscript. We will study this in the near future.
Along the same lines, although authors showed a physical interaction between NBR1 and XopL, they investigate the possibility that NBR1 also associates to XopL by means of ubiquitin chains attached to itself. Even it makes perfect sense, it is counter-intuitive to the previous observation of physical interaction. Author could try to better introduce the rational or the mechanism they hypothesize. For instance, that NBR1 is first recruited by ubiquitin chains that offer larger interaction surface (because repetitive) that leads to a subsequent direct interaction.
-Following the hypothesis that NBR1 mediates the degradation of XopL, authors test whether XopL is able to autoubiquitinate, which would potentially contribute to both its degradation, as well as the recruitment of NBR1.
Our response: We agree that the ability of NBR1 to detect and bind ubiquitin chains might be a reason that XopL is targeted by NBR1, as XopL is ubiquitinated. However, although deltaE3 lost its ability to self-ubiquitinate, and is less ubiquitinated in planta (see Fig 5H) it is still able to interact with NBR1 (see Fig 5E). This first indicated to us that the interaction occurs most likely in a ubiquitin-independent manner, which is also the case in plant-virus interactions where NBR1 binds to viral protein P4 in a ubiquitin-independent manner (Hafrén et al., 2017, PNAS). We now discuss this in L764f, p18. Nevertheless, we have tried to test interaction of XopL with different NBR1

mutants containing one or two truncations in their UBA domains, and included our results in L574-584 (Figure 5K). Briefly, full-length XopL co-immunoprecipitated with all Joka2 variants indicating that the ubiquitin-binding function of Joka2 is not needed for the interaction. However, we detected a ubiquitin-specific higher Mw smear of XopL only with Joka2 FL and ΔUBA2. Taken together, our results show that XopL and Joka2 interact in a ubiquitin-independent and dependent manner.

A previous study however, failed to show autoubiquitination of a truncated variant of XopL (singer et al. 2013). The in vivo data seems to support autoubiquitination (while adding the molecular size markers would help data interpretation, please add throughout). However, the in vitro assay is again not convincing. This may however be due to the figure which is of low quality and small (Figure S15B). Please provide a high-resolution image.
Our response: We are sorry that the image quality of FigS15B is not convincing. In Figure S16A we provide further blots showing that GST-XopL is autoubiquitinated (see extra bands in lane 1+2, absent in control lanes). The identification of "self-ubiquitination" site K191 of XopL was identified using samples that were generated within the same experiment (see Fig S16A).
3. Authors use a Luc-ATG8/NBR1 fusion to test autophagic flux and show that inoculation with Xcv, or the overexpression of XopL, result in an accumulation of both reporters. To support their claims, authors should include a positive control to show that accumulatio of the reporter is not due to overloading of the system under the used conditions, particularly by overexpression. This could be done simply by including HopM1 and/or AZD treatment.
Our response: First, we provide a positive control for autophagy suppression inf Fig S4A. Second, we do not think that overexpression of proteins in N. benthamiana results in accumulation of both reporters, as Üstün et al., 2018 show that overexpression of HopM1 leads to a reduced accumulation of the reporter and other effectors not (Fig 4A and 4B). In addition, from FigS4A in this manuscript it is obvious that overexpression of e.g., XopS or XopD are not leading to the accumulation of our reporter. XopD seems to rather decrease it.
The first XopL experiment was performed together with HopM1 in 2016 see below, where you can clearly see XopL increases the ratio while HopM1 significantly reduces the ratio.

Figure for Referee not shown.

Related to this, in Figure S4B treatment with MG132 inhibited autophagy, which is unexpected. Would the proteotoxic effect not rather activate autophagy? Please comment.
Our response: We agree that this result is somewhat surprising, as MG132 treatment is inducing proteaphagy (autophagic degradation of proteasomes). In animals it was shown that the proteasome is able to process and degrade ATG8 (Gao et al., 2010, Autophagy https://pubmed.ncbi.nlm.nih.gov/20061800/).
Using GFP-ATG8 expressing Arabidopsis plants, we can also observe that GFP-ATG8 accumulates in the presence of proteasome inhibitors or suppressors. As this is a substantial independent line of research, we feel it is beyond the scope of the current manuscript to do further analysis on this phenomenon.
4. Authors state that ATG8 levels are not further increased after ConA treatment, but surprisingly, there is rather a decrease in ATG8-labelled bodies (Figure 2C) and ATG8 (Figure 2D) protein after ConA treatment. This requires clarification.
Our response: We agree that there is a decrease of ATG8 in both assays. However, for Figure 2D it is not significant and for 2C it might be due to the fact that both XopL together with ConA might have pleiotropic effects.
5. Related to this point, ConA treatment would be expected to result in the accumulation of autophagic bodies in the vacuole. This is however not the case according to Figure 1A, or other data presented in this manuscript. Is this a N. benthamiana-specific phenomena? Please comment.
Our response: We agree that from the images in Nicotiana epidermis cells it is sometimes not easy to see whether "dots" are in the vacuole. However, in Fig S9D you can clearly see that the dots are in the vacuole (AZD/ConA condition in pTRV2 background). As Xcv and silencing of SH3P2 reduced autophagic flux into the vacuole we also do not expect huge signal in the vacuole.
6. The legend to Figure 4F is missing. Authors should also repeat this experiment, or reload the sample since there were obvious problems during the electrophoresis, which go beyond simple aesthetic problems and do not allow to confirm equal loading.

Our response: We are sorry for this mistake. As Figure 4F is "only" an expression control for the luciferase assay (Fig 4E) and we have shown expression of all of our binary constructs in other blots (Fig 1B,C,E, 2BC 4C, D), we decided to move this into the supplemental (S11C). Equal loading in this case is not essential as we only wanted to see expression of our constructs.

7. A more general point: The Rubisco band, even used commonly to asses equal loading, is utterly unappropriated for this purpose due to its inherent "overloading", because it's so abundant. Please include additional portions to include bands above and below, with less abundant bands to properly assess loading.

We have now included a supplemental figure S8E with an immunoblot against Histone H3 to asses "equal" loading when we look into SH3P2 degradation by XopL. For all of our other blots the PS is not "overloaded" you can clearly see the Rubisco Large Subunit at around 55 kDa.

Minor points

- I would suggest to simply use NBR1 and not NBR1/Joka or Joka, since NBR1 is the common and more recognized name of the protein.

We want to include both names as the role of Joka2 in immunity was first described by Dagdas et al. 2016.

-Lane #49 please include reference

Included.

-lane #469 ConA blocks vacuolar degradation, not autophagy. Please edit.

- Lane#479 Authors performed an IP not a pull-down.
- lane 697 There have been reports of effectors with autoubiquitination activity e.g. AvrPtoB form Pseudomonas

We have changed all suggestions in the revised version.

Referee #3:

The manuscript entitled "A bacterial effector counteracts host autophagy by promoting degradation of an autophagy component" reported a mutual regulatory role of autophagy in Xanthomonas-plant interaction. The authors show that a T3E XopL from Xanthomonas can suppress plant autophagy, likely by mediating the ubiquitination and degradation of a plant autophagy component SH3P2, and on the other hand, the protein level of XopL is also down-regulated by NBR1/Joka2-mediated selective autophagy. While the topic is interesting and the authors presented a large amount of data to demonstrate the molecular interplays between Xanthomonas effector and plant autophagy machinery, the interpretations of some key results are confusing and warrant clarification. Also, additional experiments are required to strengthen the major conclusions of the paper.

Specific comments:

1. Formation of autophagic structures and upregulation of ATG/NBR1 genes are usually associated with activation of autophagy, as published by previous studies including one from the corresponding author (Üstün et al., Plant Cell 2018). However, the authors claim that Xanthomonas (and later on XopL) blocks autophagy, although an increase of ATG8g-labelled autophagosomes and ATG/NBR1 expression was observed (Figure 1 and 2). This is somewhat counterintuitive. An alternative interpretation could be that Xanthomonas infection induces autophagy and XopL is one of the major inducers. There are other assays, such as detection of processed ATG8 protein or release of free GFP from GFP-ATG8 protein, that people routinely perform to indicate the activity of autophagy in planta and should be tested here during Xanthomonas infection. In Figure 1A, the increase of autophagic structures by ConA treatment (in the mock panels) seems fairly minor.

Our response: We thank the reviewer for this comment. While we agree that induction of autophagy markers might indicate that autophagy is activated, it is also evident from the literature and our own experiments that autophagy suppression also results in the same transcriptional response. As mentioned in the result section (line 167/68, p4) induced expression of autophagy marker genes is also a hallmark for autophagy suppression. This was described in (Minina et al., 2018) using autophagy deficient Arabidopsis plants. We have not scRNAseq data of the atg5 deficient mutant in which we see that all autophagy related genes are upregulated (Zhu et al., unpublished). We also provide further evidence in our own manuscript, see FigS4C: Autophagy inhibitor AIMp also leads to the induction of ATG8 as well as Joka2 (at 1dpi).

We also use the LUC-based assays that are driven by the 35S promoter and not endogenous ATG8 or NBR1 promoters. This already suggests that XopL or Xcv blocks the degradation of constitutively expressed ATG8 or NBR1 fusion proteins. In the case of NBR1 or ATG8 we can see at earlier time points (1dpi) accumulation of ATG8 or NBR1 protein levels (see Figure 1B and S3A) although transcript levels are not affected (1D, S3C). We agree that for ATG8 it is not 100% clear, but this might be due to the presence of different isoforms in N. benthamiana (6 isoforms). While ATG8-1 is not induced transcriptionally at 1dpi, ATG8-2 is induced. For the transient expression assays it is also obvious that XopL does not induce NBR1/Joka2 gene expression at 2dpi (Fig S4C), while protein levels are increased (Fig 2B). This is also additional evidence that NBR1/Joka2 autophagic degradation is impaired due to XopL-dependent autophagy suppression.

Still, we agree with the reviewer that it is possible that other effectors of Xcv might induce autophagy or autophagic degradation which is suppressed by the action of XopL. This is also subject of future research in our group.

There are other assays, such as detection of processed ATG8 protein or release of free GFP from GFP-ATG8 protein, that people routinely perform to indicate the activity of autophagy in planta and should be tested here during Xanthomonas infection.

Our response: Our lab is routinely performing the freeGFP release assay in Arabidopsis, and from many experiments we conclude that the assay does not really work in N. benthamiana (see figure below). While XopL leads to an accumulation of GFP-ATG8 fusion protein, HopM1 leads to a reduction. However, we cannot detect any freeGFP bands. We think that N. benthamiana might degrade the freeGFP moiety much faster than A. thaliana. This was also observed by Li et al., 2019, New Phytologist, as well as Dagdas et al., 2016 and Haxim et al., 2017, elife. All of these studies failed to detect any freeGFP/YFP of proteins degraded by autophagy.
All in all, we provide the quantitative luciferase assay, autophagic flux assay (-/+ConA) using confocal microscopy as well as western blot, which are in total 3 different assays to the assess autophagic response. Together with the gene expression analysis we think it is valid to conclude that XopL is suppressing autophagy.

Figure for Referee not shown.

In Figure 1A, the increase of autophagic structures by ConA treatment (in the mock panels) seems fairly minor.

Our response: The induction of autophagic structures by ConA in the mock condition is minor as this is the basal autophagy condition and we do not have any autophagy inducing conditions. This is also true for A. thaliana (see Üstün et al., 2018 Fig 4E).

Also, the inoculation approach (dipping or infiltration, at what concentration) is not clearly described in the legend. 2dpi is a relatively late point. How early can the autophagic structures be observed?

Our response: We can see increase in autophagic structures at 6-8hpi and provide a luciferase assay at 6hpi in Fig S1. We improved the legend in the revised version.

2. What is a general role of autophagy to Xcv infection? In Fig. 1E and S2A, suppression of autophagy only caused subtle difference in bacterial multiplication. This questions the importance of autophagic activity in plant-Xcv interaction. In addition, what is the phenotype of Xcv ΔxopQ ΔxopL infection under these treatments?

Our response: The effects on bacterial multiplication are significant, e.g., Fig1 we have more than 2 times more bacteria in the AIMp condition compared to WT. We have consistent effects in general autophagy (Fig1E, S2A, and SH3P2) and selective autophagy pathways (Joka2 bacterial growth). Silencing of all of these pathways contribute to bacterial multiplication. For instance, in the Joka2 VIGS bacterial growth, at 3 dpi, we have a ~0.4 log difference, meaning twice more bacteria in Joka2 VIGS. Going to the literature we see e.g., for the fls2 mutant similar growth phenotypes ((Zipfel et al., 2004; Orosa et al., 2018), which gives us confidence that the difference observed is significant.

We also must consider that we are dealing with knock down plants and not knock out mutants, which might also explain why differences are sometimes small. Moreover, silencing of Joka2 only affects a small branch of autophagy (e.g. selective degradation of ubiquitinated proteins/aggregates). The same goes for SH3P2 where autophagosome formation is impaired but they still form (see our manuscript and (Zhuang et al., 2013). This might also explain why differences are still not huge.
Regarding the phenotype we have seen slight differences most pronounced was during SH3P2 silencing (Figure 9) but usually at later time points than we measured bacterial growth. Due to its reduced growth deltaXopL generates less chlorotic symptoms in pTRV2 control plants (during bacterial growth, leaf discs were taken from leaves to measure bacterial growth). Silencing of SH3P2 slightly increases symptoms for Xcv WT and similar to the bacterial

Figure for Referee not shown.

growth increases lesions caused by deltaXopL mutant that are comparable to Xcv WT. If requested, we can add this data into the revised version.

3. The authors used different autophagy inhibitors in different assays, which is confusing. What is the difference between ConA and AIMp, in terms of inhibiting autophagy and the underlying mechanisms? A more detailed description is needed to explain why one, but not the other, is used in particular assays. This helps readers that do not directly work in the autophagy field.

Our response: We know explain this in more detail (e.g. 132f, p4). Just to further clarify:
The AIMp is a small peptide sequence derived from the PexRD54 effector that was shown to block autophagy by sequestering ATG8 proteins (Pandey et al., 2020). AIM peptide inhibits autophagosome biogenesis by occupying the binding pockets on ATG8 that mediate docking of host autophagy adaptors such as Joka2 (Pandey et al., 2021).
ConA is a drug that inhibits the V-ATPase function and thus impairs acidification of the vacuole. As vacuolar proteases work efficiently in low pH conditions, ConA treatment results in less degradation in the vacuole. This is a rather late step to interfere with general vacuolar degradation but an accepted approach in the autophagy community. Thus, we include AIMp as a very specific autophagy suppressor to complement our results.

4. Figure 3: 3B, which band indicates SH3P2-HA in the IP samples? In figure 3B-E, using SH3P2 from Arabidopsis in the interaction and protein degradation assays is odd, since all other bacteria-related assays were performed in tomato or tobacco plants. Do all SH3P2 homologs from tomato or tobacco have expression problems? What about in vitro? It is important to show the XopL interaction with and protein degradation effect on tomato or tobacco SH3P2.

Our response:
1) Not all the interaction data was performed with Arabidopsis SH3P2. The initial Y2H was done against a tobacco (Nicotiana tabacum) cDNA library and the Y2H is performed with NbSH3P2 and NtSH3P2 (this is also mentioned in the result section). The tobacco SH3P2 is 96% identical to the tomato SH3P2. The lysine 24 that is ubiquitinated by XopL (see comments referee 2 and alignment in Figure S8A) is conserved in Arabidopsis, tobacco and tomato, which indicates that the mechanisms of XopL-SH3P2 interactions are conserved. As stated in the manuscript we had problems to get expression with SH3P2 from tomato and tobacco in transient assays. 2) XopL expression in A. thaliana also leads to autophagy suppression (see Fig S7), thus we think it is legitim to work with the Arabidopsis SH3P2, as we show that this is a conserved mechanism. 3) In addition, our bacterial growth data with VIGS SH3P2 shows that the loss of XopL is compensated in SH3P2 silenced plants (Fig 3F) showing a direct contribution of NbSH3P2 to bacterial multiplication.
Unfortunately, we do not possess an antibody against SH3P2 from tobacco or tomato to perform degradation assays in these plant species.

5. Transient over-expression of bacterial effectors is prone to artifact, does XopL reduce SH3P2 protein during bacterial inoculation (e.g., comparing Xcv and XcvΔxopL mutant strain)? Is the ubiquitination level of SH3P2 protein altered during bacterial infection, comparing Xcv and XcvΔxopL mutant strain?

Our response: As mentioned above we do not possess an antibody against SH3P2 from tobacco and tomato to perform degradation assays during bacterial infection. Our bacterial growth data with VIGS SH3P2 shows that the loss of XopL is compensated in SH3P2 silenced plants (Fig 3F) showing a direct contribution of NbSH3P2 to bacterial multiplication. We think this data indirectly supports that a loss of SH3P2 has a significant positive effect during infection. Furthermore, we also show several assays (blots, luciferase assay, confocals) that the deltaXopL strain does not suppress autophagy (Fig 2D-F) to confirm the results from transient overexpression of XopL. This should demonstrate that we are not dealing with artifacts from overexpression of bacterial effectors. Additionally, we also show that by overexpression of XopL we do not see any changes of e.g., histone H3 levels, while SH3P2 is degraded (Supp Fig. 8E).
But thanks to the reviewer's remarks we decided to monitor proteins levels of SH3P2 using transiently expressed SH3P2-GFP or SH3P2-HA during infection. We do not see massive degradation of SH3P2 during infection (see Supplemental Figure 8F), because Xcv delivers a cocktail of more than 35 effectors with redundant effects/targets that might mask the function of XopL on SH3P2. Nevertheless, we can see that infection with the deltaXopL strain resulted in higher protein levels of SH3P2 for both tags, indicating that XopL is responsible for SH3P2 degradation during infection.

6. XopL has E3 ligase activity. Does it induce ubiquitination and degradation of NBR1, since they also interact and XopL, SH3P2 and NBR1 could be in the same protein complex?

Our response: As shown in Fig 2B, 5D, E and 5K, NBR1 is not degraded in the presence of XopL. XopL leads to an accumulation of NBR1 levels, thus we do not think that NBR1 is degraded by XopL in a proteasome-dependent manner.

7. Figure 5: Again, transient expression of effectors in tobacco could cause artificial results. It should be shown whether XopL protein amount is changed in NBR1-silenced/mutated plants during Xanthomonas infection.

Our response: Unfortunately, we do not have any antibody against XopL and thus are not able to provide protein levels of XopL during infection. Using a complementation strain of deltaXopL (XopL-His) is also not suitable, as it is also overexpression of an effector in bacteria using a constitutive active promoter. Another problem is that if we test this in NBR1 silenced plants we will have more bacteria multiplying in this background compared to control (see Figure 5G), which would influence the levels of XopL anyway. Sadly, the Xanthomonas community does not have any engineered Xanthomonas strains (in comparison to Pseudomonas) to study effector function during "natural" infection. We have plans to improve on this in the future.

8. NBR1/Jokes2 interacts with both GFP-XopL and GFP-XopLΔE3 (Figure 5E). Since XopLΔE3 is a point mutant of 3 amino acids, do these mutations affect its interaction with substrates? If not, NBR1/Jokes2 may also mediate the degradation of other XopL target proteins in the complex. The statements in lines 484-486 may be inappropriate.

Our response: We don't have any indication of changed interaction with substrates but show that deltaE3 is not able to degrade its target anymore. One of the initial Y2Hs was performed with a truncated version of XopL that does not have E3 ligase activity, as it lacks the c-terminal E3 ligase domain (see figure below). We would not like to include this data into our current manuscript as it is subject of future research.

Figure for Referee not shown.

Minor concerns:

1. In line 123, there is a citation with inconsistent format.
2. Line 456, Fig S5A did not show XopL protein accumulation under ConA treatment.
3. Lines 248: the "normalized RLUC-NBR1/FLUC" under Xcv ΔxopQ ΔxopL treatment seems dramatically higher than mock treatment. Therefore , "Xcv ΔxopQ ΔxopL still leads to a slight increase in both RLUC-ATG8a/FLUC and RLUC-NBR1/FLUC ratios..." may not be appropriate.

We have addressed these points in the revised version.

4. Figure S7C: the leaf shape does not look like epidermal cells. What does the purple color indicate?

Thanks for this comment, indeed the cell looks more like an Arabidopsis mesophyll cell. The purple color indicates chloroplasts. We have included a proper description in the revised version.

5. Figure 4F: the bands look ugly, especially in PS-stained blot. There is no legend for it.

We have moved this blot into the supplemental (S11C) as it is an expression control for the luciferase assay Fig4E.

Dear Suayb,

We have now received re-review reports from all three referees. They all appreciate your revisions, but referee #2 has some remaining concerns. Please provide a point-by-point response to the concerns raised.

In addition, when you submit your revised manuscript please also take care of the following points:

Please remove the figures and supplementary information from the Word file.

Please include the PRIDE identifier to your Data Availability section.

Please update the movie name to Movie EV1.

The table of contents which introduces the Appendix Figures should have page numbers; please also remove the figure and table titles and use the nomenclature "Appendix Figure S#" or "Appendix Table S#". The Appendix table should also be included in this file.

Please update the movie name to Movie EV1 and remove "Supporting Information" from the Manuscript file.

Please also upload high resolution image files.

We encourage the publication of source data, particularly for electrophoretic gels and blots, with the aim of making primary data more accessible and transparent to the reader. It would be great if you could provide me with a PDF file per figure that contains the original, uncropped and unprocessed scans of all or key gels used in the figures. The PDF files should be labeled with the appropriate figure/panel number, and should have molecular weight markers; further annotation could be useful but is not essential. The PDF files will be published online with the article as supplementary "Source Data" files. Source Data can also include Excel tables to accompany your graphs. We anticipate that their inclusion will make your work more discoverable and useable to scientists in the future.

We include a synopsis of the paper (see http://emboj.embopress.org/). Please provide me with a general summary statement and 3-5 bullet points that capture the key findings of the paper.
We also need a summary figure for the synopsis. The size should be 550 wide by [200-400] high (pixels). You can also use something from the figures if that is easier.

When preparing your letter of response to the referees' comments, please bear in mind that this will form part of the Review Process File, and will therefore be available online to the community. For more details on our Transparent Editorial Process, please visit our website: https://www.embopress.org/page/journal/14602075/authorguide#transparentprocess

Best wishes,

William

William Teale, PhD
Editor
The EMBO Journal
w.teale@embojournal.org

Given the referees' positive recommendations, I would like to invite you to submit a revised version of the manuscript, addressing the comments of all three reviewers. I should add that it is EMBO Journal policy to allow only a single round of revision, and acceptance of your manuscript will therefore depend on the completeness of your responses in this revised version.

When preparing your letter of response to the referees' comments, please bear in mind that this will form part of the Review Process File, and will therefore be available online to the community. For more details on our Transparent Editorial Process, please visit our website: https://www.embopress.org/page/journal/14602075/authorguide#transparentprocess

We generally allow three months as standard revision time. As a matter of policy, competing manuscripts published during this period will not negatively impact on our assessment of the conceptual advance presented by your study. However, we request that you contact the editor as soon as possible upon publication of any related work, to discuss how to proceed.

Thank you for the opportunity to consider your work for publication. I look forward to your revision.

Yours sincerely,

William Teale, PhD
Editor
The EMBO Journal
w.teale@embojournal.org

Use the link below to submit your revision:

https://emboj.msubmit.net/cgi-bin/main.plex

Referee #1:

The authors have addressed all the concerns that I raised in the previous round. Congratulations to all of them for an exciting story.

Referee #2:

In this new version of the manuscript "A bacterial effector counteracts host autophagy by promoting degradation of an autophagy component" Leong et al. have edited the text and added some additional data.
My main concern still stands, which is that the conclusion of a targeting of the XopL effector by the host NBR1 is not supported by the data, and authors rather following an arbitrary narrative. The fact that XopL is degraded by autophagy and that it may do so by associating to NBR1, is not a proof for their narrative. Indeed that XopL seems to interfere with autophagy through NBR1, much rather suggests the opposite, namely that XopL evolved to target NBR1 a conserved component of selective autophagy. This is also not comparable to the example in which a viral coat protein, which is conserved and rather displays traits shared with PAMPs, is targeted by NBR1.
The in vitro assays still do not convincingly show ubiquitination of SH3P2. There is an increased background in the SH3P2 lane, but no ubiquitinated forms can be recognised, not even a monoubiquitination. The bands mentioned appear randomly at distances, and not in a laddering pattern. In addition, there is basically no difference between lanes in the anti-Ub blots, except for higher molecular range in which there are changes in intensity. Nevertheless, authors do convincingly show that E3 activity of XopL is required for SH3P2 degradation, which is the main point.
Authors provide data that is not included in the manuscript to argue against this point. There are several reasons why this is problematic. First, I cannot decide on partially disclosed data, without seeing the reactions that were used, the implemented controls, and the full extent of the measurements (no. of peptides detected, coverage, quality of the spectra). Second, this does not improve data in Figure 4B. Third, readers would also not have access to this data and therefore conclusions in the text are based on data that in my view do not support them. Fourth, I do acknowledge that SH3P2 is ubiquitinated and that XopL plays a role, based on additional data provided by the authors.

Referee #3:

The authors' efforts in new experiments and clarifications are appreciated. The revised manuscript is improved. It will be good to include the Xanthomonas infection phenotype in SH3P2-silenced plant (Figure 9 in the response letter). For Supplemental Figure 8F, I assume SH3P2 is expressed under 35S promoter? It's not described in the legend. Also, the species and mutant names are not italicized in the legend.

Point-by-point response to editor and reviewers' comments on the manuscript by Leong et al.

Referee #1:

The authors have addressed all the concerns that I raised in the previous round. Congratulations to all of them for an exciting story.

We thank the referee for his encouraging words and congratulations.

Referee #2:

In this new version of the manuscript "A bacterial effector counteracts host autophagy by promoting degradation of an autophagy component" Leong et al. have edited the text and added some additional data. My main concern still stands, which is that the conclusion of a targeting of the XopL effector by the host NBR1 is not supported by the data, and authors rather following an arbitrary narrative. The fact that XopL is degraded by autophagy and that it may do so by associating to NBR1, is not a proof for their narrative. Indeed that XopL seems to interfere with autophagy through NBR1, much rather suggests the opposite, namely that XopL evolved to target NBR1 a conserved component of selective autophagy. This is also not comparable to the example in which a viral coat protein, which is conserved and rather displays traits shared with PAMPs, is targeted by NBR1.

Our response:

We provide several lines of evidence that NBR1 is targeting XopL for degradation:

- NBR1/Joka2 and XopL associate in planta 5B, C, E
- Silencing of NBR1/Joka2 leads to a stabilization of XopL in N. benthamiana (5F): the likeliest explanation for this is that NBR1 degrades XopL. If XopL targets NBR1, then VIGS-NBR1 should not result in any change in XopL abundance
- Silencing of NBR1/Joka2 result in increased susceptibility of Xcv at early stages of infection, especially when the effectors are delivered into the host cell to support bacterial multiplication
- and we provide additional data that also ubiquitinated form of XopL is recognized by NBR1/Joka2 which again argues for a targeting of XopL by NBR1/Joka2
- if XopL would target NBR1 this should result in degradation of NBR1, which we never observed. Upon expression of XopL NBR1 always accumulates (Fig. 2B, 5E). This is also true for infection with Xcv as a strain deleted in XopL does not result in an accumulation of NBR1/Joka2 (Figure 2E, S6B).
- Finally: XopL is subjected to autophagic degradation (Figure 2C, 5A, 5F, S7)

All in all, we have multiple evidence that NBR1/Joka2 is indeed targeting XopL for degradation. As mentioned in our previous rebuttal letter we agree that XopL can perturb the function of NBR1 due to the suppression of autophagy by degrading SH3P2. We also believe that other effectors from Xanthomonas (but also other bacterial pathogens) are targeted by NBR1/Joka2 and already started to work on this. However, this is is a substantial independent line of research that we feel is beyond the scope of the current manuscript. We will study this in the near future.

The in vitro assays still do not convincingly show ubiquitination of SH3P2. There is an increased background in the SH3P2 lane, but no ubiquitinated forms can be recognised, not even a monoubiquitination. The bands mentioned appear randomly at distances, and not in a laddering pattern. In addition, there is basically no difference between lanes in the anti-Ub blots, except for higher molecular range in which there are changes in intensity. Nevertheless, authors do convincingly show that E3 activity of XopL is required for SH3P2 degradation, which is the main point.
Authors provide data that is not included in the manuscript to argue against this point. There are several reasons why this is problematic. First, I cannot decide on partially disclosed data, without seeing the reactions that were used, the implemented controls, and the full extent of the measurements (no. of peptides detected, coverage, quality of the spectra). Second, this does not improve data in Figure 4B. Third, readers would also not have access to this data and therefore conclusions in the text are based on data that in my view do not

support them. Fourth, I do acknowledge that SH3P2 is ubiquitinated and that XopL plays a role, based on additional data provided by the authors.

Our response: We now provide the PRIDE identifier (in material methods section) of our proteomics experiment in which we identify XopL-dependent ubiquitination of SH3P2. This will be accessible to everyone as it also includes the in vitro ubiquitination data of XopL. Additionally, we have provided in our previous rebuttal letter the spectra of SH3P2 ubiquitination, which should be of sufficient quality (see Figure 3 in previous rebuttal letter). The experiment was conducted in triplicates (GST-XopL/MBP-SH3P2 vs. GST/MBP-SH3P2) and SH3P2 ubiquitination was only detected in GST-XopL/MBP-SH3P2 samples (this can be accessed on the PRIDE archive). However, we still do not include this data in our manuscript as this requires further research.

Referee #3:

The authors' efforts in new experiments and clarifications are appreciated. The revised manuscript is improved. It will be good to include the Xanthomonas infection phenotype in SH3P2-silenced plant (Figure 9 in the response letter). For Supplemental Figure 8F, I assume SH3P2 is expressed under 35S promoter? It's not described in the legend. Also, the species and mutant names are not italicized in the legend.

We thank the referee and we have corrected the figure legend. SH3P2 is expressed under the 35S promoter.

ACCEPTED **21st Apr 2022**

Dear Suayib,

I am pleased to inform you that your manuscript has been accepted for publication in the EMBO Journal.

Congratulations on a really great story!

Best wishes,

William

William Teale, PhD
Editor
The EMBO Journal
w.teale@embojournal.org

Please note that it is EMBO Journal policy for the transcript of the editorial process (containing referee reports and your response letter) to be published as an online supplement to each paper. If you do NOT want this, you will need to inform the Editorial Office via email immediately. More information is available here:
https://www.embopress.org/page/journal/14602075/authorguide#transparentprocess

Your manuscript will be processed for publication in the journal by EMBO Press. Manuscripts in the PDF and electronic editions of The EMBO Journal will be copy edited, and you will be provided with page proofs prior to publication. Please note that supplementary information is not included in the proofs.

Please note that you will be contacted by Wiley Author Services to complete licensing and payment information. The required 'Page Charges Authorization Form' is available here: https://www.embopress.org/pb-assets/embo-site/tej_apc.pdf

Should you be planning a Press Release on your article, please get in contact with embojournal@wiley.com as early as possible, in order to coordinate publication and release dates.

If you have any questions, please do not hesitate to call or email the Editorial Office. Thank you for your contribution to The EMBO Journal.

** Click here to be directed to your login page: https://emboj.msubmit.net

EMBO Press Author Checklist

Corresponding Author Name: Suayib Üstün	
Journal Submitted to: EMBO Journal	
Manuscript Number: EMBOJ-2021-110352R	

USEFUL LINKS FOR COMPLETING THIS FORM
The EMBO Journal - Author Guidelines
EMBO Reports - Author Guidelines
Molecular Systems Biology - Author Guidelines
EMBO Molecular Medicine - Author Guidelines

Reporting Checklist for Life Science Articles (updated January 2022)

This checklist is adapted from Materials Design Analysis Reporting (MDAR) Checklist for Authors. MDAR establishes a minimum set of requirements in transparent reporting in the life sciences (see Statement of Task: 10.31222/osf.io/9sm4x). Please follow the journal's guidelines in preparing your manuscript.

Please note that a copy of this checklist will be published alongside your article.

Abridged guidelines for figures

1. Data

The data shown in figures should satisfy the following conditions:

- the data were obtained and processed according to the field's best practice and are presented to reflect the results of the experiments in an accurate and unbiased manner.
- ideally, figure panels should include only measurements that are directly comparable to each other and obtained with the same assay.
- plots include clearly labeled error bars for independent experiments and sample sizes. Unless justified, error bars should not be shown for technical replicates.
- if n<5, the individual data points from each experiment should be plotted. Any statistical test employed should be justified.
- Source Data should be included to report the data underlying figures according to the guidelines set out in the authorship guidelines on Data Presentation.

2. Captions

Each figure caption should contain the following information, for each panel where they are relevant:

- a specification of the experimental system investigated (eg cell line, species name).
- the assay(s) and method(s) used to carry out the reported observations and measurements.
- an explicit mention of the biological and chemical entity(ies) that are being measured.
- an explicit mention of the biological and chemical entity(ies) that are altered/varied/perturbed in a controlled manner.
- the exact sample size (n) for each experimental group/condition, given as a number, not a range;
- a description of the sample collection allowing the reader to understand whether the samples represent technical or biological replicates (including how many animals, litters, cultures, etc.).
- a statement of how many times the experiment shown was independently replicated in the laboratory.
- definitions of statistical methods and measures:
  - common tests, such as t-test (please specify whether paired vs. unpaired), simple χ2 tests, Wilcoxon and Mann-Whitney tests, can be unambiguously identified by name only, but more complex techniques should be described in the methods section;
  - are tests one-sided or two-sided?
  - are there adjustments for multiple comparisons?
  - exact statistical test results, e.g., P values = x but not P values < x;
  - definition of 'center values' as median or average;
  - definition of error bars as s.d. or s.e.m.

Please complete ALL of the questions below.
Select "Not Applicable" only when the requested information is not relevant for your study.

Materials

Newly Created Materials	Information included in the manuscript?	In which section is the information available? (Reagents and Tools Table, Materials and Methods, Figures, Data Availability Section)
New materials and reagents need to be available; do any restrictions apply?	Not Applicable	

Antibodies	Information included in the manuscript?	In which section is the information available? (Reagents and Tools Table, Materials and Methods, Figures, Data Availability Section)
For **antibodies** provide the following information:<br>- Commercial antibodies: RRID (if possible) or supplier name, catalogue number and or/clone number<br>- Non-commercial: RRID or citation	Yes	Material Methods

DNA and RNA sequences	Information included in the manuscript?	In which section is the information available? (Reagents and Tools Table, Materials and Methods, Figures, Data Availability Section)
**Short novel DNA or RNA including primers, probes**: provide the sequences.	Yes	Supplemental Table 1

Cell materials	Information included in the manuscript?	In which section is the information available? (Reagents and Tools Table, Materials and Methods, Figures, Data Availability Section)
**Cell lines:** Provide species information, strain. Provide accession number in repository **OR** supplier name, catalog number, clone number, and/**OR** RRID.	Not Applicable	
**Primary cultures:** Provide species, strain, sex of origin, genetic modification status.	Not Applicable	
Report if the cell lines were recently **authenticated** (e.g., by STR profiling) and tested for mycoplasma contamination.	Not Applicable	

Experimental animals	Information included in the manuscript?	In which section is the information available? (Reagents and Tools Table, Materials and Methods, Figures, Data Availability Section)
**Laboratory animals or Model organisms:** Provide species, strain, sex, age, genetic modification status. Provide accession number in repository **OR** supplier name, catalog number, clone number, **OR** RRID.	Not Applicable	
**Animal observed in or captured from the field:** Provide species, sex, and age where possible.	Not Applicable	
Please detail **housing and husbandry conditions**.	Not Applicable	

Plants and microbes	Information included in the manuscript?	In which section is the information available? (Reagents and Tools Table, Materials and Methods, Figures, Data Availability Section)
**Plants:** provide species and strain, ecotype and cultivar where relevant, unique accession number if available, and source (including location for collected wild specimens).	Yes	Material Methods
**Microbes:** provide species and strain, unique accession number if available, and source.	Yes	Material Methods

Human research participants	Information included in the manuscript?	In which section is the information available? (Reagents and Tools Table, Materials and Methods, Figures, Data Availability Section)
If collected and within the bounds of privacy constraints report on age, sex and gender or ethnicity for all study participants.	Not Applicable	

Core facilities	Information included in the manuscript?	In which section is the information available? (Reagents and Tools Table, Materials and Methods, Figures, Data Availability Section)
If your work benefited from core facilities, was their service mentioned in the acknowledgments section?	Not Applicable	

Design

Study protocol	Information included in the manuscript?	In which section is the information available? (Reagents and Tools Table, Materials and Methods, Figures, Data Availability Section)
If study protocol has been **pre-registered, provide DOI in the manuscript**. For clinical trials, provide the trial registration number **OR** cite DOI.	Not Applicable	
Report the **clinical trial registration number** (at ClinicalTrials.gov or equivalent), where applicable.	Not Applicable	

Laboratory protocol	Information included in the manuscript?	In which section is the information available? (Reagents and Tools Table, Materials and Methods, Figures, Data Availability Section)
Provide DOI OR other citation details if **external detailed step-by-step protocols** are available.	Not Applicable	

Experimental study design and statistics	Information included in the manuscript?	In which section is the information available? (Reagents and Tools Table, Materials and Methods, Figures, Data Availability Section)

Include a statement about **sample size** estimate even if no statistical methods were used.	Yes	Figures
Were any steps taken to minimize the effects of subjective bias when allocating animals/samples to treatment (e.g. **randomization procedure**)? If yes, have they been described?	Not Applicable	
Include a statement about **blinding** even if no blinding was done.	Not Applicable	
Describe **inclusion/exclusion criteria** if samples or animals were excluded from the analysis. Were the criteria pre-established?<br><br>If sample or data points were omitted from analysis, report if this was due to attrition or intentional exclusion and provide justification.	Not Applicable	
For every figure, are **statistical tests** justified as appropriate? Do the data meet the assumptions of the tests (e.g., normal distribution)? Describe any methods used to assess it. Is there an estimate of variation within each group of data? Is the variance similar between the groups that are being statistically compared?	Yes	Figures and Material Methods

Sample definition and in-laboratory replication	Information included in the manuscript?	In which section is the information available?<br>(Reagents and Tools Table, Materials and Methods, Figures, Data Availability Section)
In the figure legends: state number of times the experiment was **replicated** in laboratory.	Yes	Figure Legend
In the figure legends: define whether data describe **technical or biological replicates**.	Yes	Figure Legend

Ethics

Ethics	Information included in the manuscript?	In which section is the information available?<br>(Reagents and Tools Table, Materials and Methods, Figures, Data Availability Section)
Studies involving **human participants**: State details of **authority granting ethics approval** (IRB or equivalent committee(s), provide reference number for approval.	Not Applicable	
Studies involving **human participants**: Include a statement confirming that **informed consent** was obtained from all subjects and that the experiments conformed to the principles set out in the WMA Declaration of Helsinki and the Department of Health and Human Services Belmont Report.	Not Applicable	
Studies involving **human participants:** For publication of **patient photos**, include a statement confirming that consent to publish was obtained.	Not Applicable	
Studies involving experimental **animals**: State details of **authority granting ethics approval** (IRB or equivalent committee(s), provide reference number for approval. Include a statement of compliance with ethical regulations.	Not Applicable	
Studies involving **specimen and field samples:** State if relevant **permits** obtained, provide details of authority approving study; if none were required, explain why.	Not Applicable	

Dual Use Research of Concern (DURC)	Information included in the manuscript?	In which section is the information available?<br>(Reagents and Tools Table, Materials and Methods, Figures, Data Availability Section)
Could your study fall under dual use research restrictions? Please check biosecurity documents and list of **select agents and toxins** (CDC): https://www.selectagents.gov/sat/list.htm	Not Applicable	
If you used a select agent, is the security level of the lab appropriate and reported in the manuscript?	Not Applicable	
If a study is subject to dual use research of concern regulations, is the name of the **authority granting approval and reference number** for the regulatory approval provided in the manuscript?	Not Applicable	

Reporting

The MDAR framework recommends adoption of discipline-specific guidelines, established and endorsed through community initiatives. Journals have their own policy about requiring specific guidelines and recommendations to complement MDAR.

Adherence to community standards	Information included in the manuscript?	In which section is the information available?<br>(Reagents and Tools Table, Materials and Methods, Figures, Data Availability Section)
State if relevant guidelines or checklists (e.g., **ICMJE, MIBBI, ARRIVE, PRISMA**) have been followed or provided.	Not Applicable	
For **tumor marker prognostic studies,** we recommend that you follow the **REMARK** reporting guidelines (see link list at top right). See author guidelines, under 'Reporting Guidelines'. Please confirm you have followed these guidelines.	Not Applicable	
For **phase II and III randomized controlled trials**, please refer to the **CONSORT** flow diagram (see link list at top right) and submit the CONSORT checklist (see link list at top right) with your submission. See author guidelines, under 'Reporting Guidelines'. Please confirm you have submitted this list.	Not Applicable	

Data Availability

Data availability	Information included in the manuscript?	In which section is the information available?<br>(Reagents and Tools Table, Materials and Methods, Figures, Data Availability Section)
Have **primary datasets** been deposited according to the journal's guidelines (see 'Data Deposition' section) and the respective accession numbers provided in the Data Availability Section?	Yes	Proteomics Data will be deposited on PRIDE.
Were **human clinical and genomic datasets** deposited in a public access-controlled repository in accordance to ethical obligations to the patients and to the applicable consent agreement?	Not Applicable	
Are **computational models** that are central and integral to a study available without restrictions in a machine-readable form? Were the relevant accession numbers or links provided?	Not Applicable	
If publicly available data were reused, provide the respective **data citations in the reference list**.	Not Applicable	
